# FAK displacement from focal adhesions: a promising strategy to target processes implicated in cancer progression and metastasis

**DOI:** 10.1186/s12964-020-00671-1

**Published:** 2021-01-07

**Authors:** Ioanna Antoniades, Maria Kyriakou, Anna Charalambous, Katerina Kalalidou, Andri Christodoulou, Maria Christoforou, Paris A. Skourides

**Affiliations:** Department of Biological Sciences, University of Cyprus, P.O. Box 20537, 2109 Nicosia, Cyprus

**Keywords:** Focal adhesion kinase (FAK), Paxillin, Focal adhesions, Cell migration, Cell invasion, Cancer

## Abstract

**Background:**

Focal adhesion kinase (FAK) is a non-receptor tyrosine kinase that is overexpressed or activated in several advanced-stage solid cancers. It is known to play both kinase-dependent and -independent roles in promoting tumor progression and metastasis. Numerous inhibitors, targeting either the enzymatic or scaffolding activities of FAK have been generated, with varying degree of success. Here, we describe a novel approach to site-specifically target both kinase-dependent and -independent FAK functions at focal adhesions (FAs), the primary sites at which the kinase exerts its activity.

**Methods:**

We took advantage of the well-characterized interactions between the paxillin LD motifs and the FAK FAT domain and generated a polypeptide (LD2-LD3-LD4) expected to compete with interactions with paxillin. Co-immunoprecipitation experiments were performed to examine the interaction between the LD2-LD3-LD4 polypeptide and FAK. The effects of LD2-LD3-LD4 in the localization and functions of FAK, as well as FA composition, were evaluated using quantitative immunofluorescence, cell fractionation, FA isolation and Western Blot analysis. Live cell imaging, as well as 2-D migration and cell invasion assays were used to examine the effects on FA turnover and tumor cell migration and invasion.

**Results:**

Expression of the LD2-LD3-LD4 polypeptide prevents FAK localization at FAs, in a controlled and dose-dependent manner, by competing with endogenous paxillin for FAK binding. Importantly, the LD2-LD3-LD4 peptide did not otherwise affect FA composition or integrin activation. LD2-LD3-LD4 inhibited FAK-dependent downstream integrin signaling and, unlike existing inhibitors, also blocked FAK’s scaffolding functions. We further show that LD2-LD3-LD4 expression markedly reduces FA turnover and inhibits tumor cell migration and invasion. Finally, we show that dimers of a single motif, linked through a flexible linker of the proper size, are sufficient for the displacement of FAK from FAs and for inhibition of tumor cell migration. This work raises the possibility of using a synthetic peptide as an antimetastatic agent, given that effective displacement of FAK from FAs only requires dimers of a single LD motif linked by a short flexible linker.

**Conclusion:**

In conclusion, these results suggest that FAK displacement from FAs is a promising new strategy to target critical processes implicated in cancer progression and metastasis.

Video abstract.

**Graphical abstract:**

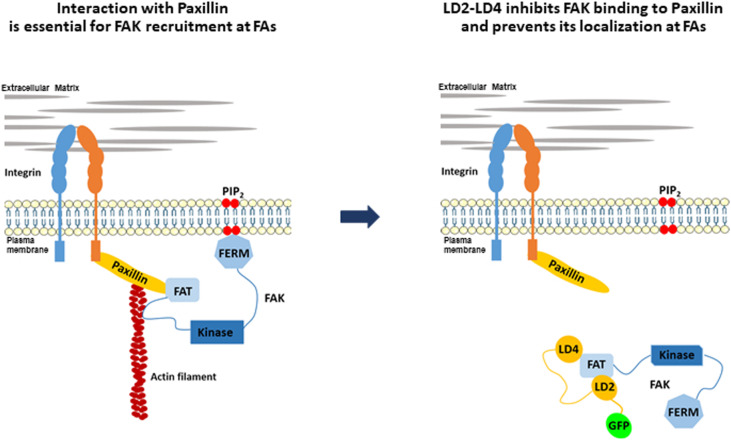

**Supplementary Information:**

**Supplementary information** accompanies this paper at 10.1186/s12964-020-00671-1.

## Background

Cells and their associated microenvironment exhibit a complicated bidirectional communication that is critical for both normal tissue homeostasis and for tumor cell growth, progression and metastasis [1]. The Focal Adhesion Kinase (FAK) has been identified as a critical regulator and signal transducer for these Extracellular Matrix (ECM)-tumor cell interactions [2, 3] and has been implicated in many aspects of the metastatic process including adhesion, migration, secretion of MMPs, spindle orientation and invasion [4–7]. FAK has also been found to exhibit altered (usually elevated) expression and/or activation in most human epithelial cancers, resulting in enhanced invasive potential and poor overall patient survival [8]. FAK has therefore, become an attractive target for anti-cancer therapies.

FAK has kinase-dependent and -independent functions, both of which are involved in cancer progression. However, most of the recently developed inhibitors, have focused on blocking the protein’s enzymatic activity. These include antibodies [9, 10], dominant negative constructs [11–14] and small molecule inhibitors, that primarily target the ATP binding site, or allosterically inhibit the kinase domain [15], leading to decreased tumor cell viability, growth or apoptosis. However, targeting the kinase domain of FAK has been complicated by the fact that the ATP-binding site shares consensus sequences and structural domains across many different tyrosine kinases, making it less suitable for clinical testing, due to off-target effects [16].

An alternative approach is to inhibit FAK’s kinase-independent activities by blocking specific scaffolding functions of the protein. This has been attempted using peptides, small molecule and antibody inhibitors that disrupt interactions between FAK and various binding partners including VEGFR-3 [17], IFGR1 [18], c-Met [19, 20], Mdm-2 [21] and p53 [22], with variable efficiency.

One of the most highly studied multi-protein complexes, that serve as sites of integration of growth factor signaling and integrin pathways, directing changes as diverse as gene expression and cytoskeletal reorganization, are Focal Adhesions (FAs). The focal adhesion targeting (FAT) domain of FAK is both necessary and sufficient for localization at FAs and facilitates interactions with FA-associated proteins including Paxillin [23, 24], Talin [25, 26], p130Cas [27], Grb2, ASAP1 [28] and p85α of PI3K [29]. It is a highly conserved four helix bundle with a large hydrophobic core [30, 31]. It includes two surface exposed hydrophobic pockets (HPs), one located at the surface of helices 1 and 4, and the second located at the surface of helices 2 and 3 [32]. These are absolutely essential for FAK targeting to FAs [30, 33, 34]. The best characterized interaction of the FAT HPs is that with the short (9 amino acids) Leucine-rich LD motifs of Paxillin [23, 24]. This interaction is important for FAK FA localization, suggesting that Paxillin is one of the major proteins responsible for the kinase’s recruitment to these complexes [33–35]. Paxillin itself, is targeted to FAs via the C-terminal LIM (Lin11, Isl-1 & Mec-3) domains, however the interacting proteins responsible for its localization have not been identified [24, 36]. Once FAK is localized at FAs, it phosphorylates various proteins either directly or through the recruitment of Src kinases [37, 38], leading to efficient integrin signal transduction and cell migration.

The aim of the present study was to develop and test a new strategy for the simultaneous inhibition of both enzymatic and scaffolding functions of FAK, specifically at FAs, by interfering with FAK targeting to these complexes. To do so, we took advantage of the well characterized binding of the LD motifs of Paxillin with the FAK FAT HPs, to prevent interactions with endogenous proteins, responsible for FA targeting. We show that a polypeptide including the LD2 and LD4 motifs of Paxillin, specifically displaces endogenous FAK from FAs in a dose dependent manner without otherwise affecting FA composition or integrin activation. We go on to show that these effects are the result of a competing interaction of the polypeptide with endogenous paxillin, for binding to the FAT domain of FAK. Furthermore, we show that effective displacement of FAK from FAs can be accomplished using dimers of a single LD motif, linked by a short flexible linker. FAK displacement from FAs leads to inhibition of downstream integrin signaling, reduced FA turnover, defects in cell spreading and inhibition of cell migration and invasion. Our findings demonstrate that preventing FAK targeting and functions, specifically at FAs, represents a promising new strategy to prevent molecular and cellular processes implicated in tumor cell metastasis.

## Materials and methods

### Plasmids and DNA constructs

#### FLAG LD2-LD3-LD4

The DNA encoding amino acids 54–279 of human Paxillin, was amplified via PCR using pCS108 GFP LD2-LD3-LD4 as template and primers F2 and R2 (Additional File 1: Table S1).

The PCR program was as follows: 2 min at 95 °C for initial denaturation, followed by 35 cycles of 15 s at 95 °C, 30 s at 67 °C, 1 min at 68 °C and final extension at 68 °C for 10 min.

The PCR product was cloned in frame and downstream to FLAG, in pFLAG CMV-2, using the NotI and EcoRI restriction sites.

#### GFP LD2-LD3-LD4 and mCherry LD2-LD3-LD4

The DNA encoding amino acids 54–279 of human Paxillin, was amplified via PCR using pCS2++ GFP-Paxillin as template and primers F1 and R1 (Additional File 1: Table S1).

#### GFP LD2-LD3-LD4 ΔLR

The DNA encoding amino acids 139–279 of Paxillin, was amplified via a two-step PCR using GFP LD2-LD3-LD4 as template and primers F3, F4 and R2 (Additional File 1: Table S1).

#### GFP LD2-LD4

For the generation of GFP LD2-LD4 constructs with linkers of various sizes, multi-step PCRs using the following templates and primers were performed.

GFP LD2-LD4 30aa (referred in the text as GFP LD2-LD4): GFP LD2-LD3-LD4 ΔLR was used as template and primers F4, R4, R5, R6 and R3 (Additional File 1: Table S1).

GFP LD2-LD4 15aa: GFP LD2-LD4 30aa was used as template and primers F4, R7 and R3 (Additional File 1: Table S1).

GFP LD2-LD4 25aa: GFP LD2-LD4 15aa was used as template and primers F4, R8 and R3 (Additional File 1: Table S1).

#### GFP LD2

The DNA encoding amino acids 420–449 of human Paxillin, was amplified via PCR using pCS2++ GFP LD2-LD4 as template and primers F5 and R9 (Additional File 1: Table S1).

#### GFP LD4

The DNA encoding amino acids 783–845 of human Paxillin, was amplified via PCR using pCS2++ GFP LD2-LD4 as template and primers F6 and R10 (Additional File 1: Table S1).

The PCR program for the above was as follows: 2 min at 95 °C for initial denaturation, followed by 35 cycles of 15 s at 95 °C, 30 s at 67 °C, 1 min at 68 °C and final extension at 68 °C for 10 min.

The PCR products of the above were cloned into a pCS108 vector (between NotI and XhoI restriction sites) already including either the EGFP or mCherry gene (between EcoRI and NotI).

#### GFP LD2-LD2

A two-step cloning strategy was followed using GFP LD2-LD4 6x short as template.

An *LD2-linker* fragment was generated using primers F4, R11 and R12 (Additional File 1: Table S1).

The PCR product was cloned in frame and downstream to GFP, in pCS108-GFP using the NotI and XbaI restriction sites.

A *linker-LD2* fragment was generated using primers F7, F8 and R13 (Additional File 1: Table S1):

The PCR product was cloned in frame in the pCS108-GFP LD2-linker plasmid, using the XbaI and XhoI restriction sites.

#### GFP LD4-LD4

A multi-step PCR was performed, using GFP LD2- LD4 as template and primers F9, F10, R14, R15 and R12 (Additional File 1: Table S1):

The PCR program was as follows: 2 min at 95 °C for initial denaturation, followed by 35 cycles of 15 s at 95 °C, 30 s at 67 °C, 1 min at 68 °C and final extension at 68 °C for 10 min.

The PCR product was cloned in frame and downstream to GFP, in pCS108-GFP using the NotI and XhoI restriction sites.

#### pLV-tetO-LD2-LD4.

The DNA encoding motifs LD2 and LD4 connected with a 30 amino acid-long linker composed of 6 GGGS repeats was amplified via PCR using pCS108 GFP LD2-LD4 as template and primers F11 and R16 (Additional File 1: Table S1). The PCR program was as follows: 2 min at 95 °C for initial denaturation, followed by 35 cycles of 15 s at 95 °C, 30 s at 67 °C, 1 min at 68 °C and final extension at 68 °C for 10 min. The PCR product was cloned in pLV-tetO-Oct4 vector, using the EcoRI restriction site (to replace Oct4) [39].

pCS2++ TagRFP FAK, pCS108 FusionRed Vinculin and pCS108 RFP Vinculin were generated by replacing the GFP sequence with that of TagRFP or FusionRed in pCS2++ GFP FAK and pCS108 GFP Vinculin respectively [40]. pCS2++ mKate FAK was described elsewhere [34]. pCS2-myc-GFP-dSH2 was obtained from Addgene.

### Cells, cell culture and transfection

HeLa (CCL-2) and MDA MB-231 (HTB-26) cells were obtained from ATCC and were tested for mycoplasma contamination. HeLa and MDA MB-231 cells were maintained in DMEM (Biosera) supplemented with 10% FBS (Biosera) and 1X Antibiotic-Antimycotic (Gibco). H460 (HTB-177) cells were maintained in RPMI 1640 medium (ThermoFisher Scientific) with 10% FBS, 1 mM sodium pyruvate (Gibco) and 1X Antibiotic-Antimycotic. HCT 116 (CCL-247) cells were maintained in McCoy’s 5A medium (ThermoFisher Scientific) supplemented with 10% FBS and 1X Antibiotic-Antimycotic. Transient transfections with Lipofectamine 2000 (Invitrogen) were performed according to manufacturer’s instructions. Cells were observed 24 h after transfection to verify expression and then used for subsequent experiments.

### GFP and GFP LD2-LD4 stable cell line generation

For the production of infectious viral particles, HEK 293 T cells were transfected (calcium phosphate) with lentiviral plasmids encoding either a) reverse tetracycline-controlled trans-activator (rtTA-N144) (Addgene), or b) GFP LD2-LD4 (pLV-tetO-LD2-LD4) or c) GFP (pLenti-CMV-GFP-Hygro) (Addgene), together with the packaging (pCMV-dR8.91) and envelope (pCMV-VGV-G) plasmids. Viral supernatants were collected after 48 and 72 h, filtered using a 0.45 mm syringe and stored at 4^ο^ C.

GFP Hela cells were generated by transduction of pLenti-CMV-GFP-Hygro, while GFP LD2-LD4 Hela cells were generated by co-transduction of pLV-tetO-LD2-LD4 and rtTA-N144 viral supernatants, in the presence of 10 μg/ml Polybrene (Merck Millipore). 10 μg/ml doxycycline was added for 48 h to induce GFP-LD2-LD4 expression [41]. Cells were allowed to recover for 72 h prior to selection with hygromycin B (Sigma-Aldrich) (400 μg/ml for 10 days).

### Antibodies

Antibodies used for western blot analysis: mouse anti-GFP (1:1000; Proteintech, #50430-2-AP), rabbit anti-pFAK Y576 (1:200; Santa Cruz, #sc-16563), rabbit anti-pFAK Y397 (1:200; Novus, #NBPI-60837), rabbit anti-pPaxillin Y31 (1:200; Santa Cruz, #sc-14035), mouse anti-FAK (1:1000; Proteintech, #66258-I-1g), mouse anti-Paxillin (1:5000; BD Biosciences, #610051), rabbit anti-FLAG (1:1000; Proteintech, #80010-1-RR).

Antibodies used in immunofluorescence experiments: mouse anti-FAK 4.47 (1:1000; Millipore, #05-537), mouse anti-FAK (1:1000; Proteintech, #66258-I-1g), rabbit anti-FAK C-20 (1:200; Santa Cruz, #sc-558), rabbit anti-pFAK Y576 (1:200; Santa Cruz, #sc-16563), rabbit anti-pFAK Y397 (1:200; Novus, #NBPI-60837) goat anti-Talin C-20 (1:750; Santa Cruz, #sc-7534), mouse anti-Paxillin (1:750; R&D Systems, #AF4259), mouse anti-Vinculin (1:1000; Sigma, #V9131), rabbit anti-Vinculin (1:2000; Proteintech, #26520-I-AP), mouse anti-av Integrin H-2 (1:100; Santa Cruz, #sc-376156), rabbit anti-pPaxillin Y31 (1:200; Santa Cruz, #sc-14035), mouse anti-p-Tyr (pY20) (1:200; Santa Cruz, #sc-508), rat anti-active β1-Integrin 9EG7 (1:700; BD Bioscences, #550531), mouse anti-p130 Cas 35B.1A4 (1:500; Santa Cruz, #sc-20029), mouse anti-Tensin C-2 (1:1000; Santa Cruz, #sc-376367).

### Focal adhesion isolation

The isolation of focal adhesion complexes was based on the protocol of Jones et al. (2015) with some modifications [42]. Briefly, cells were plated on 10-cm cell culture dishes and grown for 48 h (10 plates per condition). Cells were fixed with 1% formaldehyde for 5 min at room temperature and formaldehyde was quenched with 0.125 M glycine for 10 min. Cells were washed with ice cold PBS and incubated with modified RIPA buffer for 5 min on ice. Cell bodies were removed with high-pressure water and the remaining focal adhesion were collected by scraping in Laemmli sample buffer.

### Cell Lysis, Western blot and Immunoprecipitation

For western blot analysis cells were rinsed with ice-cold PBS and lysed in RIPA buffer supplemented with protease inhibitors (2x Halt Protease inhibitor cocktail, Thermo Scientific) and sodium orthovanadate (5 mM, Sigma) for 10 min. Lysates were cleared by centrifugation (15,000 g, 4 °C for 10 min) and 40 μg of extracted protein were used for Western blot analysis as previously described [34].

For GFP or FLAG pull-down, cells from two 10-cm plates for each condition, were lysed in 1.5 ml lysis buffer (20 mM Tris pH 7.5, 150 mM NaCl, 1% v/v NP40) supplemented with protease inhibitors and sodium orthovanadate. Cleared protein extracts were gently mixed with 5 μl of GFP-Trap Agarose beads (Chromotek) or 30 μl anti-FLAG M2-Agarose affinity gel (Sigma Aldrich), overnight at 4 °C. The beads or gel were washed 3 times with lysis buffer and then resuspended in 50 μl 2x Laemli sample buffer (Bio-Rad) supplemented with 2-mercaptoethanol (Fluka). Samples were boiled for 10 min at 95 °C and the supernatant containing the immunocomplexes was collected by centrifugation (2500 g, 4 °C for 5 min) and analysed by western blot.

For FAK immunoprecipitation, five 10-cm plates of GFP, uninduced GFP LD2-LD4 or induced LD2-LD4 cells were lysed in 1.5 ml lysis buffer (as described above). Protein extracts were incubated with 10 μg of anti-FAK antibody (Proteintech), overnight at 4 °C (extracts of uninduced GFP LD2-LD4 cells incubated with no antibody were used as a negative control.). Extracts were then incubated with 30 μl Protein-G Sepharose CL-4B beads (GE Healthcare) for 2 h at 4 °C. Beads were washed 5 times with lysis buffer and boiled in 40 μl 2x Laemli sample buffer and analysed by western blot.

### Inhibitor treatment

Cells were incubated at 37 °C with either 50 μM Chloropyramine hydrochloride (C4) inhibitor (Santa Cruz Biotechnology) for 24 h, or 10 μM PF228 inhibitor (Santa Cruz Biotechnology) for 3 h.

### Immunofluorescence

Cells (~ 7 × 10^4^) seeded on HCl-charged glass coverslips (15 mm), were PFA or methanol/acetone fixed and immunostained as described elsewhere [40]. Briefly, cells were rinsed with cold PBS and fixed with 4% PFA for 10 min, followed by quenching with 10 mM Glycine (Sigma) for 10 min and permeabilization with 0.2% Triton X-100 (Bio-Rad) for 10 min. Alternatively cells were fixed with cold methanol/acetone (1:1) for 20 min at − 20 °C. Cells were then blocked with 10% donkey serum (Jackson Immumoresearch) in 1X PBS for 30 min and incubated with primary antibodies for 1 h. This was followed by several washes in 1X PBS and incubation with secondary antibodies for 1 h. Cells were thoroughly washed with 1X PBS and mounted in ProLong Diamond antifade mountant (Molecular Probes).

### Invasion assay

25–30,000 MDA MB-231 cells, transiently transfected with LD2-LD3-LD4, were resuspended in a solution of 30% Matrigel (Corning), 0.02% Hoechst (Invitrogen) and 1% FBS in DMEM (final volume: 20 μl) and placed as a droplet on a rectangular chambered coverslip (24x60mm), previously treated with organosilane (RainX). A round 10 mm coverslip, with 2 mm spacers was placed on top, flattening the droplet into a round disc. The Matrigel was allowed to set in a humidifying chamber (37 °C) for 30 min. A second layer of 30% Matrigel (with 5% FBS) containing 0,02% fluorescent beads (Molecular Probes) (final volume: 50 μl), was added under the round coverslip, to occupy the area surrounding the disc and allowed to set as described above. The chambered rectangular coverslip was then filled with DMEM (containing 10% FBS). The setup was used for time-lapsed imaging of cell invasion over a period of 48–72 h. Alternatively, it was used to obtain static images after 48 or 72 h.

### Imaging

Confocal imaging was performed on a Zeiss LSM 710 laser scanning confocal microscope (Carl Zeiss AG, Germany), with a Plan-Apochromat 63x/1.40 oil DIC immersion objective using lasers 488 nm, 543 nm and 633 nm. Super-resolution imaging was performed on a Zeiss LSM 900 laser scanning confocal microscope with Airyscan 2 (Carl Zeiss AG, Germany) with a Plan-Apochromat 63x/1.40 oil DIC immersion objective using lasers 475 nm, 555 nm, 630 nm. Widefield fluorescence imaging was performed on a Zeiss Axio Imager Z1 microscope (Carl Zeiss AG, Germany), with a Plan-Apochromat 63x/1.40 oil Ph3 immersion objective. Live cell migration imaging was performed on a Zeiss Axio Imager Z1 microscope and an Axiovert 200 motorized inverted microscope (Carl Zeiss AG, Germany), with a Plan-Apochromat10x/0.4 Ph1 objective. Western blot imaging was performed using a UVP Biospectrum imaging system.

Image analysis was performed using Zen 2010 and Axiovision 4.8 software (Carl Zeiss AG, Germany) and Adobe Photoshop (Adobe Inc., San Jose CA) to adjust brightness and contrast. Figures were constructed with Adobe Photoshop.

### Quantification

VisionWorks software (UVP LLC, CA) was used for the quantification of western blot results using raw data from non-processed images for densitometry analysis.

Imaris image analysis software (Oxford Instruments, UK) and Zen 2010 software (Carl Zeiss AG, Germany) were used for the quantification of fluorescence intensities. Classification was according to GFP expression levels and was performed by determining the median GFP intensity of expressing cells in each experiment and comparing this to the GFP intensity of individual cells from the same experiment. Non-expressing cells were used as negative controls and were intrinsic to each experiment, since transient transfections never lead to 100% transfection efficiency.

To quantify FA-localization of specific proteins, cells were stained for the protein of interest as well as an additional FA marker, shown to retain its localization upon LD2-LD3-LD4 expression (i.e. vinculin or talin). Individual FAs were automatically selected, based on the staining of the unaffected FA marker, and the mean cytosolic intensity of each protein was subtracted from that on individually selected FAs, thus enabling specific protein quantification on individual FAs. To quantify the localization of the Src SH2 domain at FAs, cells were transfected with GFP Src dSH2 alone or together with mCherry LD2-LD3-LD4. Cells were stained for Vinculin and individual FAs were selected based on Vinculin staining. FA localization was then assessed as described above. For the quantification of FA turnover rates, time-lapse images of cells expressing RFP Vinculin (used as an FA marker) were aligned in ImageJ (NIH, USA) and analyzed. Individual FAs were manually selected (ROI) at time point t0 and the mean fluorescence intensity of each ROI was documented for each time point of the recording (t0 = 0 min, t1 = 5 min, t2 = 10 min, t3 = 15 min, t4 = 20 min, t5 = 25 min). The mean cytosolic intensity for each time point was subtracted from that of each ROI.

Quantification of cell migration rates was performed using the Imaris image analysis software and performing automatic tracking of individual cells. The migration efficiency was calculated as the percentage change of the migration rate of GFP expressing cells, compared to that of non-expressing control cells.

Quantification of cell invasion efficiency was performed with the Imaris image analysis software, using static images of cells at the end of 72 h. Total cell numbers were determined by automatic selection of individual nuclei (stained with Hoechst). LD2-LD3-LD4 expressing cell numbers were determined by automatic selection of GFP positive cells. Control cell numbers were determined by subtracting GFP positive cell numbers from total cell numbers. Invading cell numbers were determined by automatic detection of cells within a specific ROI. ROIs included areas defined by a fluorescently delineated boundary between a fluorescently-labeled low-serum concentration gel and a non-labeled high-serum concentration gel. The invasion efficiency was calculated as the percentage of cells that traversed the boundary.

### Statistical analysis

Graph generation and statistical analysis were performed using Prism software (GraphPad, San Diego, CA). All graph data are shown as mean values while error bars represent S.E.M. Statistical analysis was performed using two-tailed unpaired t-tests with 95% confidence interval. For experiments examining FAs, all statistical analysis was performed on total FA numbers from all cells considered. All experiments have been performed at least 3 times.

## Results

### Using the Paxillin LD motifs to interfere with interactions targeting FAK to FAs, as a strategy to inhibit FAK

**Fig. 1:**
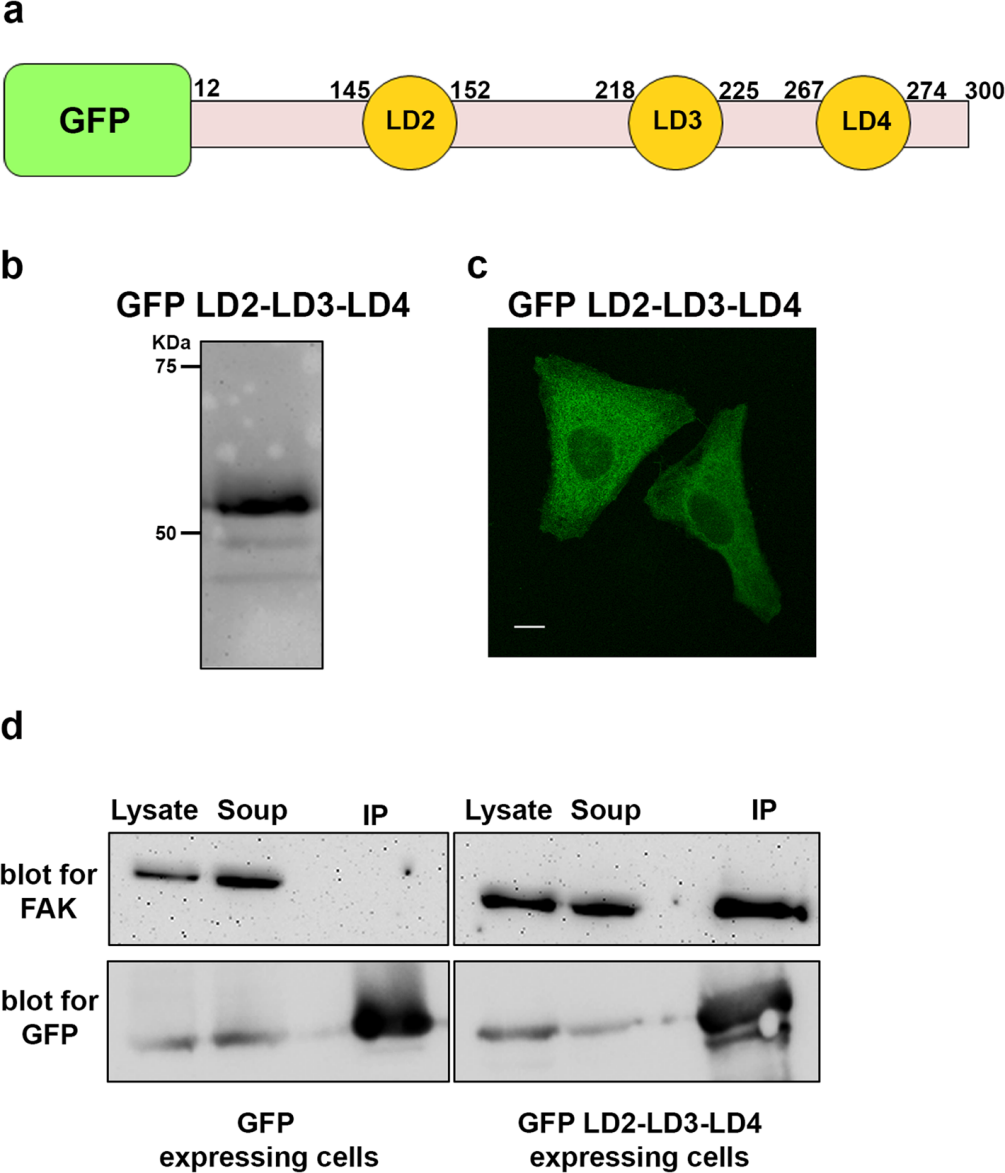
LD2-LD3-LD4 interacts with FAK directly **a**) Schematic representation of the LD2-LD3-LD4 polypeptide, composed of amino acids 54–279 of Paxillin, including the LD2-LD3-LD4 motifs and fused to GFP. **b**) Representative Western Blot (using anti-GFP) showing expression of a stable protein encoding GFP-fused LD2-LD3-LD4, in HeLa cells (expected molecular weight ~ 51 kDa). **c**) Confocal images of PFA-fixed HeLa cells expressing GFP LD2-LD3-LD4. The protein is primarily localized in the cytosol (Scale bar: 10 μm). **d**) Western blots showing immunoprecipitated GFP (left) and GFP LD2-LD3-LD4 (right), blotted for GFP and FAK. Co-precipitation of FAK (125 kDa) is only observed in HeLa cells co-expressing GFP LD2-LD3-LD4

Our main aim was to develop and test a new strategy that would block both enzymatic and scaffolding functions of FAK, specifically at FAs, as a possible new approach for FAK inhibition. To achieve this, we set out to interfere with interactions responsible for FA targeting. The FAT domain is both necessary and sufficient to drive FAK at FAs. Previous work from our group and others revealed that the two hydrophobic pockets (HPs) formed by the FAT domain are essential for FA targeting [33, 34, 43]. The best characterized interaction of the HPs is with the LD2 and LD4 motifs of Paxillin [30, 35]. We postulated that a peptide encoding these LD motifs, but lacking FA targeting sequences (LIM domains), would interfere with interactions responsible for FAK FA targeting. We also took into account the fact that several cancer-linked Paxillin mutations have been mapped to the intrinsically disordered regions between LDs and not on the motifs themselves, such as P30S, G105A and A127T that lie between LD1 and LD2 and P233L and T255I that lie between LD3 and LD4 [44, 45]. Given the significance of this intermediate linking region, in LD interactions with binding partners and in LD scaffolding functions, we decided that it should be included in the construct [46]. We therefore generated a construct containing LD2-LD3-LD4 and intermediate linking regions, fused to GFP, hereunto referred to as LD2-LD3-LD4 (Fig. 1a). This construct led to expression of a stable protein, at the expected molecular weight, which localized primarily in the cytosol (Fig. 1b and c).

We went on to examine if LD2-LD3-LD4 interacted with FAK, in co-immunoprecipitation experiments, using extracts of HeLa cells transiently transfected with GFP LD2-LD3-LD4 or GFP (negative control). As shown in Fig. 1d, a band corresponding to co-precipitated FAK (at 125kD), was detected only in the precipitates from cells expressing GFP LD2-LD3-LD4, showing that LD2-LD3-LD4 specifically interacts with FAK, as expected. Overall, these experiments show that LD2-LD3-LD4 interacts with FAK directly and given its cytosolic localization it could potentially prevent FAK localization at FAs.

### Expression of LD2-LD3-LD4 leads to the specific and dose-dependent displacement of FAK from FAs

**Fig. 2:**
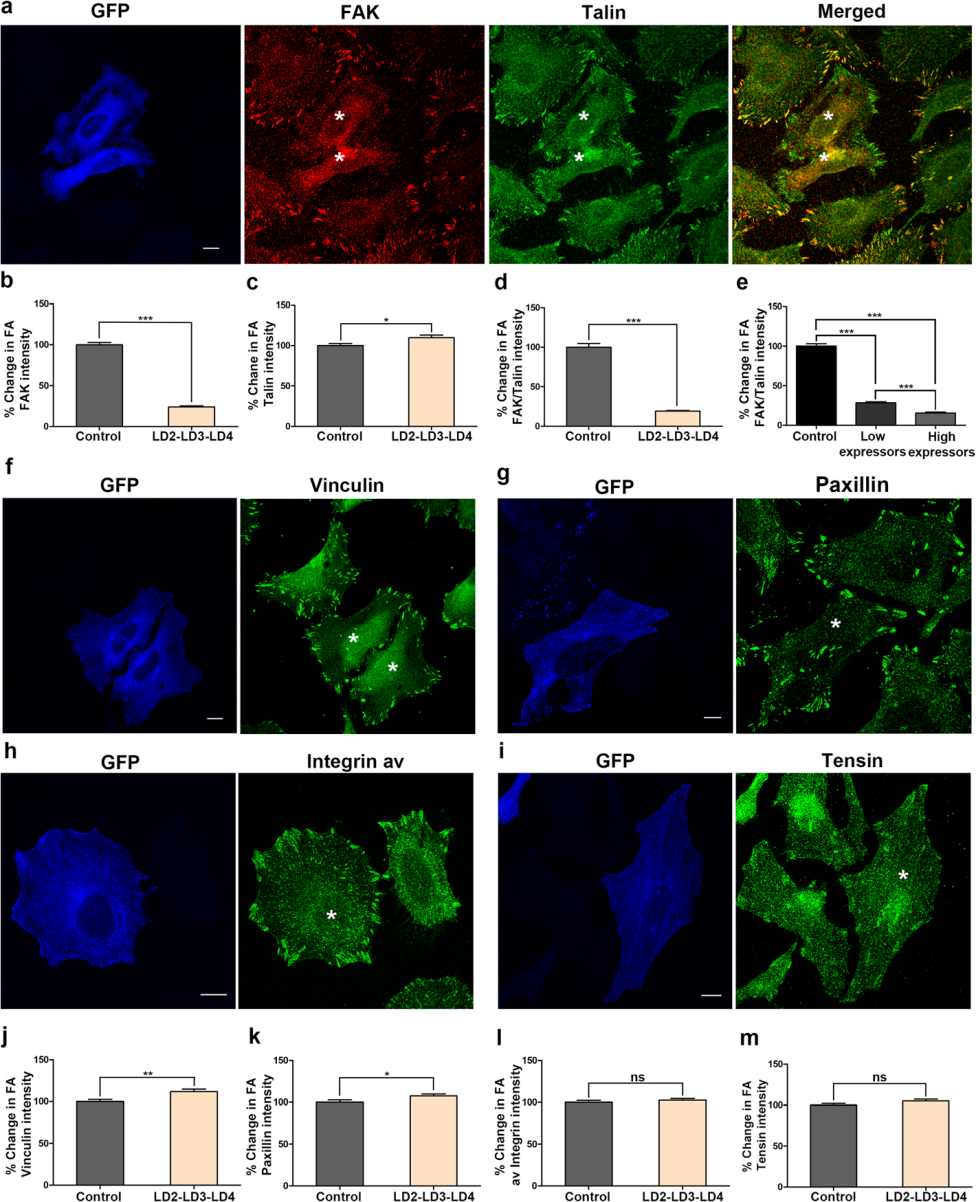
Expression of LD2-LD3-LD4 leads to the dose-dependent displacement of FAK from FAs without affecting overall FA composition **a**) Confocal images of methanol/acetone fixed HeLa cells, transiently transfected with GFP LD2-LD3-LD4 and immunostained for FAK and Talin. In control cells, FAK strongly localizes at FAs, labeled with Talin. In cells expressing LD2-LD3-LD4 (marked with asterisk) there is no detectable enrichment of FAK at FAs. In contrast, Talin localization at FAs is unaffected. **b**-**c**) Quantification of the % change in the mean FA/cytosolic FAK (**b**) and Talin (**c**) intensity ratios. FA/Cytosolic ratio of FAK in control cells is ~ 4.2 fold higher (100 ± 2.86, *n* = 469 FAs from 30 cells) compared to cells expressing GFP LD2-LD3-LD4 (23.78 ± 1.25, *n* = 409 FAs from 30 cells), indicating that LD2-LD3-LD4 leads to the displacement of FAK from FAs. In contrast, the FA/cytosolic ratio of Talin is higher in cells expressing GFP LD2-LD3-LD4, possibly due to increased FA size (109.7 ± 3.22 in expressing, compared to 100 ± 2.59 in controls). **d**) Quantification of the % change in the mean FAK/Talin intensity (based on **b** and **c**) reveals a ~ 5-fold drop of FA localized FAK in cells expressing GFP LD2-LD3-LD4 (19.01 ± 0.91 in expressing, compared to 100 ± 4.8 in control cells). **e**) Dose-dependent displacement of FAK from FAs as indicated by quantification of the % change in the mean FAK/Talin intensity in control compared to expressing cells (high and low) (15.55 ± 1.25, *n* = 188 FAs from 13 cells, expressing higher, and 28.52 ± 1.14, *n* = 218 FAs from 17 cells, expressing lower amount of GFP LD2-LD3-LD4, compared to 100 ± 3.1, *n* = 229 FAs from 28 control cells). To discriminate between high and low expressing cells, we initially determined the mean GFP intensity of all expressing cells, and then compared this to the GFP intensity of individual cells, so as to classify them as high or low expressors. (**f**-**i**) Confocal images of PFA-fixed control and GFP LD2-LD3-LD4 (marked with asterisk) HeLa cells immunostained for Vinculin (**f**), Paxillin (**g**), av. Integrin (**h**) and Tensin (**i**) showing that localization of these proteins is not affected by the expression of GFP LD2-LD3-LD4. (**j**-**m**) Corresponding quantification of the % change in the mean FA/cytosolic intensity for each protein presented in f-i. FA/cytosolic ratio of Vinculin (**j**) and Paxillin (**k**) is higher in cells expressing GFP LD2-LD3-LD4, possibly due to increased FA size (111.8 ± 2.86, *n* = 460 FAs from 30 expressing, compared to 100 ± 2.57, *n* = 455 FAs from 30 control cells for Vinculin; 107.5 ± 2.29, *n* = 402 FAs from 30 expressing, compared to 100 ± 2.64, *n* = 432 FAs from 30 control cells for Paxillin). There is no significant difference in the FA/cytosolic ratio of av. Integrin (**l**) and Tensin (**m**) (102.5 ± 1.94, *n* = 568 FAs from 30 expressing, compared to 100 ± 2.16, *n* = 485 FAs from 30 control cells for av. Integrin; 105 ± 2.24, *n* = 532 FAs from 30 expressing, compared to 100 ± 2.09, *n* = 568 FAs from 30 control cells for Tensin). Scale bars: 10 μm. The error bars represent standard error of the mean (S.E.M). ***; *p* < 0.0001, **; *p* < 0.005, *; *p* < 0.05

In order to examine if LD2-LD3-LD4 expression could specifically disrupt FAT domain interactions and displace FAK from FAs, HeLa cells were transiently transfected with LD2-LD3-LD4, seeded on FN coated coverlips for two hours, fixed and immuno-stained for FAK and Talin. Talin was selected as a stable marker of mature FAs, given the fact that its recruitment to the complex relies on direct binding to β integrin cytoplasmic tails. As shown, expression of LD2-LD3-LD4 led to the clear displacement of FAK from FAs, while Talin localization was unaffected (Fig. 2a). This effect was confirmed using a second FAK antibody and, in addition, exogenous mKate FAK (Additional File 2: Fig. S1 a-c). We quantified this displacement, by calculating the ratio of FAK in the cytosol to FAK at FAs, revealing that LD2-LD3-LD4 expression led to a 4-fold reduction of FA-localized FAK (Fig. 2b). Interestingly, a similar quantification for Talin showed that LD2-LD3-LD4 expression leads to an increase in FA localized Talin, possibly due to enlargement of the FA complexes (Fig. 2c). In order to account for this, we calculated the FAK to Talin ratio at FAs, which revealed a dramatic 5-fold reduction in LD2-LD3-LD4 expressing cells, suggesting that LD2-LD3-LD4 is very effective in displacing FAK from FAs (Fig. 2d), unlike expression of GFP, which was used as a negative control (Additional File 2: Fig. S1d). We then examined how the levels of LD2-LD3-LD4 affected displacement efficiency, and revealed a clear dose response relationship; in cells expressing relatively high levels of GFP, we observed complete loss of FAK from FAs while in cells expressing moderate or low levels of GFP, we could still detect FAK at FAs, albeit at significantly reduced levels (Fig. 2e).

Fluorescent proteins, despite mutations to reduce their ability to dimerize, still maintain some capacity to do so. Additionally, given their globular nature and relatively large size (27kD) they tend to stabilize fused peptides. Furthermore, GFP displays inherent accumulation to the nucleus and could thus be influencing peptide function, by affecting cellular distribution. To ensure that the LD2-LD3-LD4 peptide is stable and can be used effectively in the absence of GFP, we generated a FLAG- tagged peptide which is much smaller in size (1kD). As shown in Fig. S1e and S1f, the FLAG-tagged peptide can efficiently interact with FAK and lead to effective displacement from FAs., This confirms that the LD2-LD3-LD4 is sufficiently stable and functional in the absence of a large globular protein.

A previously generated inhibitor of the interaction of FAK with VEGFR3 (C4), was also reported to displace FAK from FAs [47]. This interaction, as characterized by docking studies, takes place through binding of C4 to His 1025 on Helix 4 of the FAT domain of FAK, adjacent to HP1, to which it may sterically hinder access [17]. We thus decided to compare the efficiency of C4 with that of LD2-LD3-LD4, to displace FAK from FAs. We examined the distribution of FAK and Talin in HeLa cells, following treatment with high concentrations of C4 (50 μM) for 48 h. Surprisingly, C4, failed to visibly displace FAK from FAs, compared to controls. Quantification of FAK to Talin signal ratios on FAs of control and treated cells, confirmed that C4 did not affect FAK FA localization. However, after inhibitor treatment, some cells appeared to have smaller FAs, with low FAK and Talin signals, because they were detaching from the substrate. These data show that C4 does not specifically block FAK targeting to FAs and suggest that in order to efficiently displace the protein, disruption of interactions taking place at the HPs is necessary (Additional File 2:Fig. S1g).

Given the potent displacement of FAK from FAs induced by LD2-LD3-LD4, we wanted to examine the specificity of this effect and possible consequences on FA composition. We therefore examined the localization of additional core FA proteins including Integrins (av), Paxillin, Tensin and Vinculin. As shown, similarly to Talin, FA localization of these proteins was not reduced by LD2-LD3-LD4 expression, suggesting that the effect of LD2-LD3-LD4 is specific to FAK and that the composition of FA complexes is broadly unaltered in expressing cells (Fig. 2f-m). Overall, these data provide evidence that LD2-LD3-LD4 could serve as an effective, site-specific inhibitor of interactions at the HP sites within the FAT domain of FAK and prevent FAK localization at FAs in a dose-dependent manner.

### LD2-LD3-LD4 inhibits both kinase-dependent and scaffolding functions of FAK at FAs

**Fig. 3:**
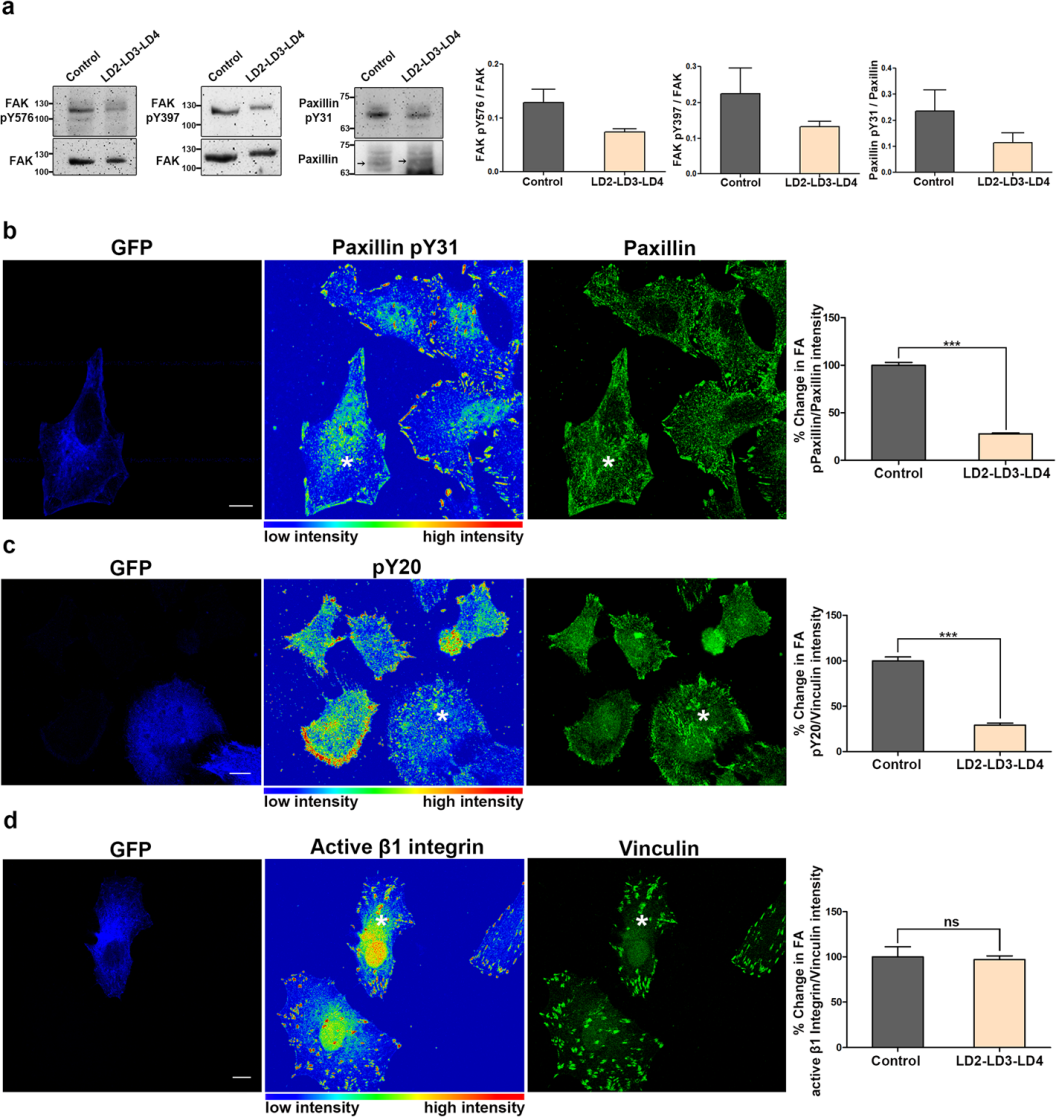
Expression of LD2-LD3-LD4 blocks kinase-dependent functions of FAK, downstream of integrin activation **a**) Representative Western Blots and quantification from control cells and cells expressing GFP LD2-LD3-LD4, indicating the phosphorylation status of FAK Tyr 397 and Tyr 576 and Paxillin Tyr 31. Quantification of the ratio of phosphorylated FAK over total FAK shows reduction of the phosphorylation at both Tyr 576 (0.13 ± 0.024 in control, 0.074 ± 0.005 in GFP LD2-LD3-LD4 expressing samples) and Tyr 397 (0.22 ± 0.0073 in control, 0.13 ± 0.015 in GFP LD2-LD3-LD4 expressing samples). Paxillin Tyr 31 phosphorylation is also reduced, as indicated by the ratio of phosphorylated over total paxillin (0.24 ± 0.08 in control, 0.11 ± 0.04 in GFP LD2-LD3-LD4 expressing samples). **b**) Confocal images of PFA-fixed HeLa cells transfected with GFP LD2-LD3-LD4 and immunostained for Paxillin and phosphorylated Paxillin (pY31). Phosphorylated Paxillin signal in control and GFP LD2-LD3-LD4 expressing cells (marked with asterisk) is presented in the middle panel as an intensity color-coded image. Quantification of the % change in the mean intensity of phosphorylated (pPaxillin) to total Paxillin reveals significant reduction of Paxillin phosphorylation at FAs in cells expressing GFP LD2-LD3-LD4 (27.93 ± 0.83, *n* = 331 FAs from 24 cells) compared to control cells (100 ± 2.92, *n* = 371 FAs from 25 cells). **c**) Confocal images of PFA-fixed HeLa cells transfected with GFP LD2-LD3-LD4, immunostained against phosphorylated tyrosine (pY20) and Vinculin. Intensity of tyrosine phosphorylation in control and GFP LD2-LD3-LD4 expressing cells (marked with asterisk) is presented in the middle panel in a color-coded image. Quantification of the % change in the mean pY20/Vinculinintensity reveals a 3.4-fold decrease in total phosphorylation at FAs in cells expressing GFP LD2-LD3-LD4 (29.29 ± 2.12, *n* = 414 FAs from 30 cells) compared to control cells (100 ± 4.44, *n* = 435 FAs from 30 cells). **d**) Confocal images of PFA-fixed cells transfected with GFP LD2-LD3-LD4 and immunostained against active β1 Integrin and Vinculin. Active β1 Integrin signal in control and GFP LD2-LD3-LD4 expressing cells (marked with asterisk) is presented in the middle panel as an intensity color-coded image. Quantification of the % change in the mean active β1 Integrin/Vinculin intensity shows that expression of GFP LD2-LD3-LD4 does not affect integrin activation at FAs (100 ± 11.18, *n* = 346 FAs from 30 control, compared to 97.10 ± 3.98, *n* = 457 FAs from 30 expressing cells). Scale bars: 10 μm. The error bars represent standard error of the mean (S.E.M). ***; *p* < 0.001

FAK is a major transducer of integrin signaling and becomes phosphorylated and activated in response to integrin-dependent adhesion. Given that LD2-LD3-LD4 interacts with FAK directly, leading to its displacement from FAs, we went on to address its effects on FAK activation. To do so, we examined the phosphorylation state of a) Tyr397, the major FAK auto-phosphorylation site required for activation, b) Tyr576, which resides in the activation loop of the kinase domain and has been shown to lead to full activation upon phosphorylation and c) paxillin Tyr31, one of the major FAK/Src downstream targets [37, 48]. As shown, LD2-LD3-LD4 expression led to a significant reduction of phosphorylation at these sites, suggesting that LD2-LD3-LD4 expression blocks FAK activation and downstream signaling (Fig. 3a). Importantly, this reduction becomes even more significant, since transient transfection efficiency is never 100% and thus what we observe represents an underestimation of the effect. In order to examine the effects of LD2-LD3-LD4 on FAK phosphorylation in individual cells, we carried out Immunofluorescence (IF) using phospho-specific antibodies. As shown, LD2-LD3-LD4 expression led to a dramatic drop in FAK phosphorylation (on Tyr397) at FAs, suggesting that it effectively eliminates FAK activation at these complexes (Additional File 2: Fig. S1h).

One of the best characterized downstream targets of FAK is Paxillin, which becomes phosphorylated on Tyrosines 31 and 118, in response to integrin activation in wild type but not in FAK null cells [49]. We thus went on using quantitative immunofluorescence and calculated the ratio of phosphorylated-Paxillin (pPax) to Paxillin, in order to assess the effects of LD2-LD3-LD4 on Paxillin phosphorylation, specifically at FAs. As shown, expression of LD2-LD3-LD4 led to a significant reduction of the levels of pPax, suggesting that it not only blocks FAK activation but also downstream signaling from FAs (Fig. 3b). In agreement with this result, staining of LD2-LD3-LD4 expressing cells with a well characterized pY antibody (pY20) revealed that overall tyrosine phosphorylation is dramatically reduced at FAs, suggesting that signaling is impaired due to FAK displacement (Fig. 3c). Given the dramatic reduction of tyrosine phosphorylation at FAs, we examined whether LD2-LD3-LD4 somehow prevents integrin activation. Quantification of the ratio of active Integrin β1 to Vinculin at FAs showed that LD2-LD3-LD4 has no effect on integrin activation (Fig. 3d). Therefore, the above data clearly show that LD2-LD3-LD4 expression blocks FAK kinase-dependent signal transduction events, downstream of integrin activation.

**Fig. 4:**
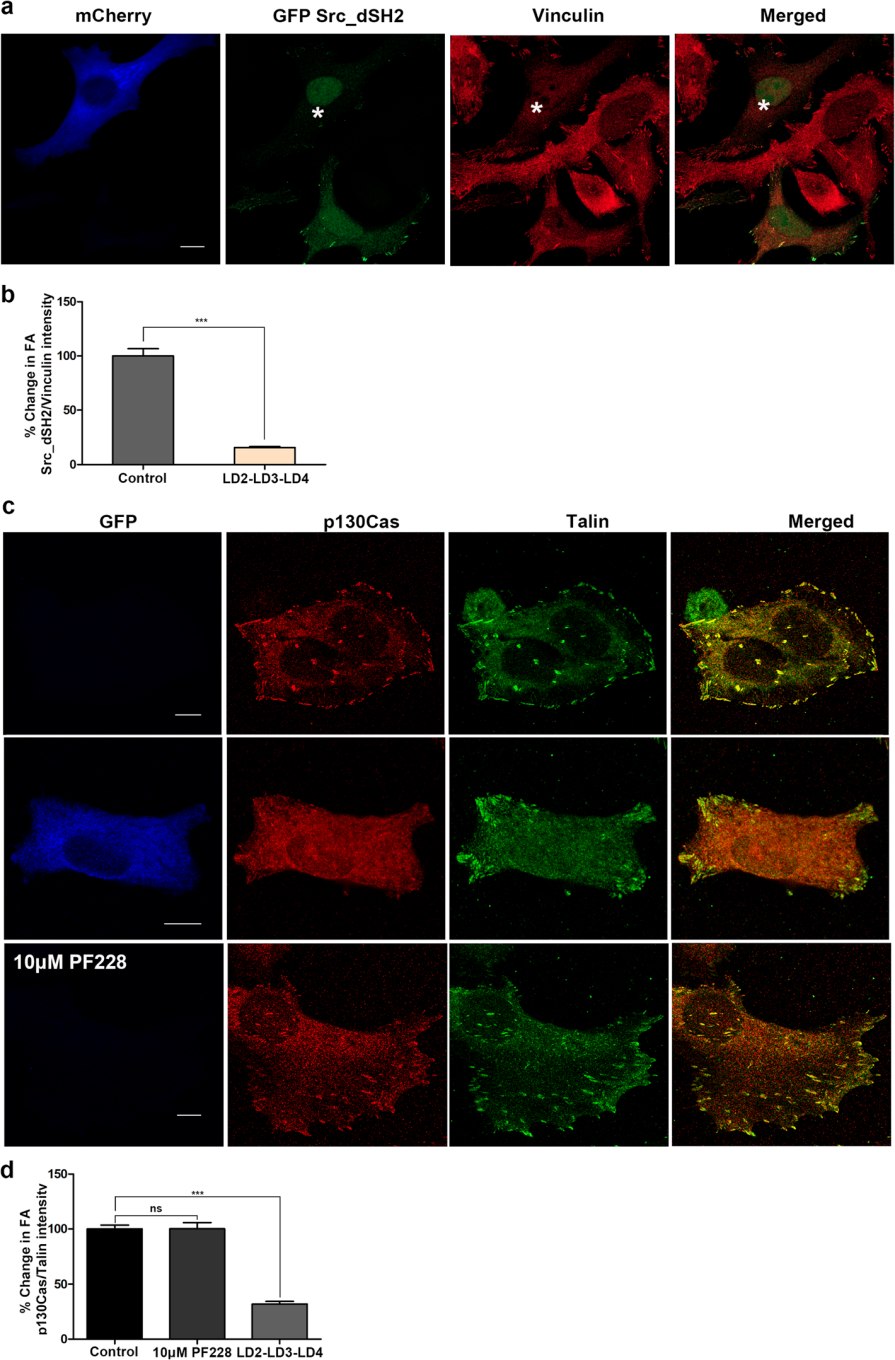
Expression of LD2-LD3-LD4 blocks kinase-independent, scaffolding functions of FAK at F**As a**-**b**) Super resolution images and quantification of the FA-localization of GFP Src_dSH2 in control (GFP Src_dSH2 only) and mCherry LD2-LD3-LD4 expressing cells (marked with asterisk). Cells were fixed with PFA and immunostained for Vinculin. Expression of mCherry LD2-LD3-LD4 leads to 84.3% decrease in the Src_dSH2/Vinculin ratio compared to control cells (100 ± 6.68, *n* = 419 FAs from 24 control, 15.67 ± 0.93, *n* = 373 FAs in 22 mCherry LD2-LD3-LD4 expressing cells). **c**-**d**) Confocal images (**c**) and quantification (**d**) of control (top panel), LD2-LD3-LD4 expressing (intermediate panel) and PF228 treated (bottom panel) cells, fixed with methanol/acetone and immunostained for p130Cas and Talin. Expression of LD2-LD3-LD4 leads to a 68% decrease in the p130Cas/Talin ratio compared to control cells, whereas treatment with PF228 does not elicit any significant change (100 ± 3.56, *n* = 505 FAs from 33 control, 31.89 ± 2.46, *n* = 564 FAs from 35 LD2-LD3-LD4 expressing and 100.2 ± 5.6, *n* = 515 FAs from 30 PF228-treated cells). F Scale bars: 10 μm. The error bars represent standard error of the mean (S.E.M). ***; *p* < 0.0001

Upon recruitment at FAs, FAK is auto-phosphorylated on Tyr397, creating a high-affinity binding site for the SH2 domain of Src, which further phosphorylates FAK on Tyr576 and Tyr577 within the activation loop, leading to maximal enzymatic activity [50]. In order to examine the effects of LD2-LD3-LD4 expression on FAK-mediated Src recruitment to FAs we expressed the SH2 domain of Src fused to GFP (GFP Src_dSH2), previously shown to be necessary and sufficient for FA targeting of Src [51]. As expected, given the previous data indicating FAK FA displacement and abolishment of Tyr397 phosphorylation, expression of LD2-LD3-LD4 led to a significant reduction in Src_dSH2 FA localization, indicating an inability of Src to target FAs (Fig. 4 a and b).

FAK also has well-established scaffolding functions, including a kinase independent role in the recruitment of the FAK-Src substrate, p130Cas to FAs [27]. This is achieved through an SH3-dependent interaction with the C terminal proline-rich regions of FAK [27]. In order to determine if, unlike kinase inhibitors, LD2-LD3-LD4 could also suppress kinase-independent, scaffolding functions, we examined p130Cas localization. LD2-LD3-LD4 expressing and control cells, as well as cells treated with a previously characterized FAK kinase inhibitor (PF228) [52], were immunostained for Talin and p130Cas. There was a visible reduction of FA-localized p130Cas in LD2-LD3-LD4 expressing cells, unlike control and PF228-treated cells in which no change was observed as confirmed by quantification of the ratio of p130Cas to Talin (Fig. 4c and d). As expected PF228 treatment led to a clear reduction of tyrosine phosphorylated FAK at FAs but did not interfere with its localization; thus, as expected, p130Cas is maintained at the complex (Additional File 2: Fig. S2a-c). The above results show that unlike inhibitors of FAK’s enzymatic activity, expression of LD2-LD3-LD4 blocks both kinase-dependent and independent functions at FAs.

### Expression of LD2-LD3-LD4 affects FA dynamics, and inhibits migration and invasion of tumor cells

**Fig. 5:**
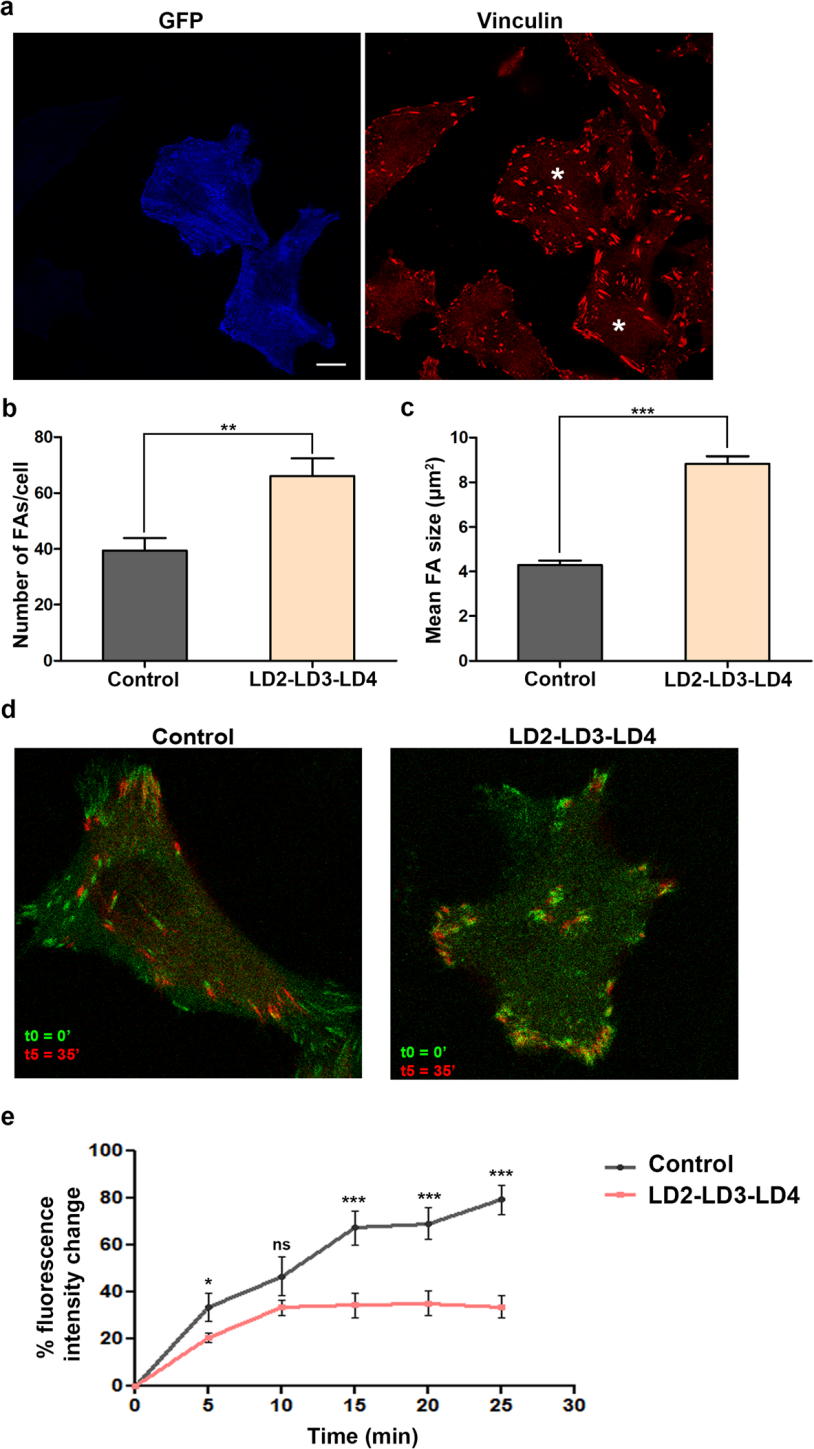
Expression of LD2-LD3-LD4 leads to an increase in number and size of FAs and a reduction of FA turnover **a**) Confocal images of PFA-fixed HeLa cells transfected with GFP LD2-LD3-LD4 and immunostained for Vinculin. Cells expressing GFP LD2-LD3-LD4 (marked with asterisk) form more and larger FAs, that are localized more ventrally. (**b**-**c**) Quantification of the total number of FAs per cell (**b**) and mean FA size (**c**), confirm significant increase of FA number (39.41 ± 4.48 FAs, *n* = 27 control cells compared to 66.17 ± 6.35 FAs, *n* = 29 GFP LD2-LD3-LD4 expressing cells) and size (4.29 ± 0.21 μm^2^, *n* = 526 FAs from 27 control cells, compared to 8.83 ± 0.33 μm^2^, *n* = 915 FAs from 29 GFP LD2-LD3-LD4 expressing cells) and. d) Overlayed confocal images from time lapse recordings, showing FA turnover in control and GFP LD2-LD3-LD4 expressing cells. Cells were imaged over a period of 25 min and FAs were visualized using RFP Vinculin. **e**) Quantification of the FA turnover rate assessed as the percentage change in the average intensity of selected FAs over time. FAs in GFP LD2-LD3-LD4 expressing cells display significantly slower turnover rates (33.77% ± 4.86 change at 25 min, n = 52 FAs) compared to control cells (79.39% ± 6.22change at 25 min, *n* = 38 FAs). **; *p* < 0.005, ***; *p* < 0.0001, *; *p* = 0.0118, ***; *p* ≤ 0.0005

It is well established that FAK is a critical regulator of FA assembly and disassembly, processes that are fundamental for efficient, directional cell migration [53–55]. Given that expression of LD2-LD3-LD4 displaces FAK from FAs, we initially examined whether this would elicit changes in FA dynamics. For this purpose, we evaluated the number and size of FAs in LD2-LD3-LD4 expressing vs control HeLa cells that were seeded on glass coveslips for 12 h. As shown in Fig. 5a, control cells formed a characteristic pattern of FAs, mainly found at the cell periphery. In contrast, LD2-LD3-LD4 expressing cells displayed a significant increase in both the number and size of FAs with prominent ventral FAs (Fig. 5b and c). This result, suggests a defect in FA turnover and is consistent with previous findings in FAK −/− cells [53]. We went on to directly examine the effects of LD2-LD3-LD4 expression on FA turnover. HeLa cells were transfected with RFP-Vinculin alone or co-transfected with RFP-Vinculin and LD2-LD3-LD4, seeded on fibronectin-coated chambered slides and time-lapse sequences were recorded, over a period of 35 min. Cells expressing the construct displayed markedly slower FA turnover compared to control cells (Fig. 5d and e). Therefore, these data show that LD2-LD3-LD4 expression elicits defects in FA turnover, leading to the appearance of more and larger FAs, in a similar manner to defects reported in FAK null fibroblasts [53].

**Fig. 6:**
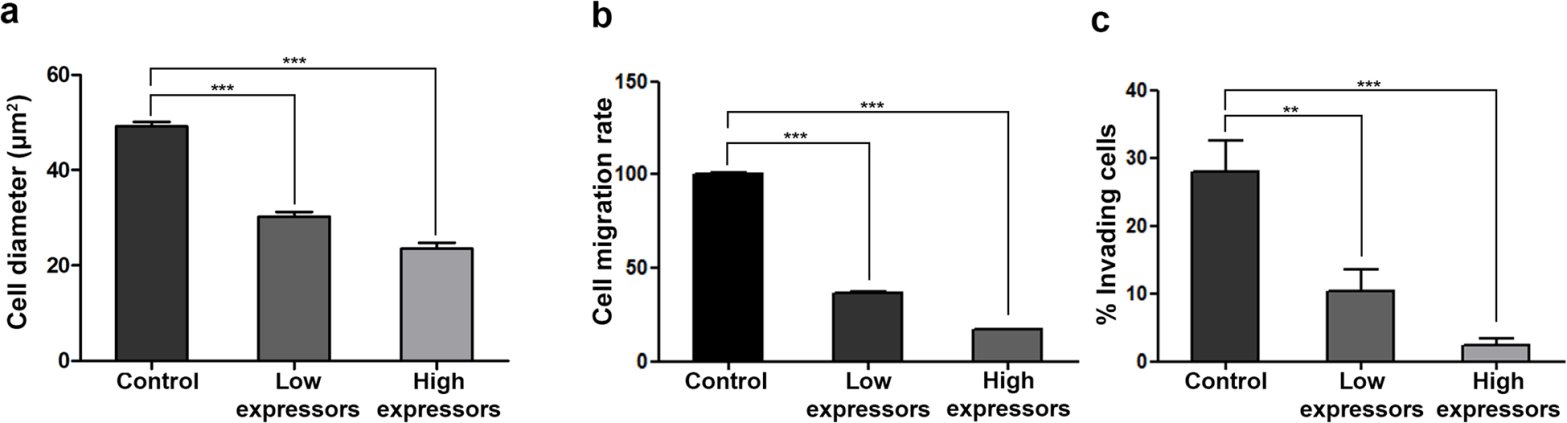
LD2-LD3-LD4 expression inhibits tumor cell spreading, migration and invasion in a dose-dependent manner **a**) Expression of GFP LD2-LD3-LD4 leads to dose-dependent defects in Hela cell spreading. Spread area is reduced by 38.4 and 52.2% in low-and high-expressing cells respectively (49.16 ± 0.97 μm, *n* = 216 control cells, compared to 30.27 ± 0.92 μm, *n* = 102 low-expressing cells and 23.52 ± 1.28 μm, *n* = 67 high-expressing cells). Cell spreading was calculated as a function of the diameter of the attached area on fibronectin coated coverslips, 1 h after seeding. **b**) Expression of GFP LD2-LD3-LD4 leads to dose-dependent defects in migration of HeLa. The rate of migration is reduced by 63 and 83% in low and high-expressing cells respectively, following 16 h of recording (100 ± 1.32, *n* = 549 in control, compared to 36.62 ± 0.28, *n* = 263 in low-expressing and 17.03 ± 0.2, *n* = 227 in high-expressing cells). **c**) Expression of GFP LD2-LD3-LD4 leads to a dose-dependent reduction in the capacity of MDA-MB231 cells to invade Matrigel, as indicated by quantification, using Hoechst staining to detect all cells and GFP to determine expressors. Invasion efficiency is reduced by 63 and 92% in low-and high-expressing cells respectively, following 72 h of incubation (27.95 ± 4.5%, n = 22,718 control, compared to 10.39 ± 03.06%, *n* = 8950 low-expressing and 2.3 ± 1.02%, *n* = 5930 high-expressing cells). Invasion efficiency is calculated as the percentage of cells traversing a fluorescently delineated boundary from a low- to high-serum concentration gel, in a chemotactic gradient. To discriminate between high and low expressing cells, we initially determined the mean GFP intensity of all expressing cells, and then compared this to GFP intensity of individual cells, so as to classify them as high or low expressors. ***; *p* < 0.0001, **; *p* < 0.005


Given the central role of FA turnover in cell migration, we decided to examine how LD2-LD3-LD4 affected cell spreading and migration. Control and LD2-LD3-LD4 expressing HeLa cells were seeded on fibronectin-coated coverslips and monitored using time-lapse video microscopy over a period of 16 h. We used a motorized stage to image multiple areas simultaneously, so as to record and track large numbers of cells. Analysis of the recordings revealed that LD2-LD3-LD4 elicited dose-dependent defects in both cell spreading and migration (Fig. 6a and b and Additional File 3: Movie S1). In addition, analysis of the time-lapse images revealed that cells expressing high levels of LD2-LD3-LD4, displayed slightly increased apoptosis (16,9% compared to 6,2% in control cells). Similar effects were observed in other highly migratory and metastatic cell lines, namely MDA MB-231 (Additional File 2: Fig. S3a), H460 (Additional File 2: Fig. S3b) and HCT-116 (Additional File 2: Fig. S3c), in which FAK is effectively displaced from FAs, upon expression of LD2-LD3-LD4 (Additional File 2: Fig. S3d-f). Overall, these data show that LD2-LD3-LD4, not only elicits defects in cell spreading and FA turnover, consistent with phenotypes observed in FAK null cells, but is also an effective inhibitor of two-dimensional (2-D) cell migration.

**Additional file 3: Movie S1.** Expression of LD2-LD3-LD4 reduces the migration of HeLa cells.

Although active cell migration is a prerequisite for metastasis, there is strong evidence suggesting that 3-D culture and gel invasion assays better mimic the tumor microenvironment and predict therapeutic responses, in vivo*,* more accurately [56, 57]. To examine the effects of LD2-LD3-LD4 on tumor cell invasion, we developed a modified Boyden-chamber gel invasion assay, which allows live and end-point evaluation of cell invasion and permits imaging, tracking and quantification of both invading and non-invading cells. The highly invasive MDA MB-231 cells were used for these experiments and both end-point measurements, as well as time-lapse recordings were generated with mixed populations of LD2-LD3-LD4 expressing and control cells, in the same setup. As shown, LD2-LD3-LD4 expression, inhibited invasion of MDA MB-231 cells in a dose-dependent manner; in fact, high expression drastically reduced invasion of this highly metastatic cell line (Fig. 6c and Additional File 4: Movie S2). These results show that displacement of FAK from FAs is an effective strategy to block both cell migration, as well as tumor cell invasion. It could therefore form the basis for the development of anti-metastatic drugs.


**Additional file 4: Movie S2.** Expression of LD2-LD3-LD4 inhibits the invasion of MDA MB-231 cells.

### The LD2 and LD4 motifs are sufficient for effective FAK displacement from FAs

Previous work revealed that LD2 and LD4 are responsible for the interaction with the HPs of FAK [30, 35]. In the work described above, the construct used to displace FAK from FAs also contained LD3, as well as intermediate linking regions (Fig. 1a). This was initially deemed necessary given the significant regulatory role assigned to these unstructured linking segments for the FAK-paxillin interaction [44–46]. However, these regions also bare numerous phosphorylation sites and binding sites for proteins other than FAK, thus their presence would be expected to be detrimental to the specificity of the polypeptide and lead to off target effects. In addition, the large size of the polypeptide containing these regions (24 kDa, 226aa) poses restrictions in its potential use as a metastatic inhibitor, in the form of a synthetic peptide since it is well beyond the size limit for effective peptide synthesis (100-120aa). Therefore, we decided to determine the minimum paxillin sequences required for efficient displacement of FAK from FAs. To this end we implemented a subtractive approach, removing individual linking regions in a stepwise fashion and assessing the activity of each construct.

**Fig. 7:**
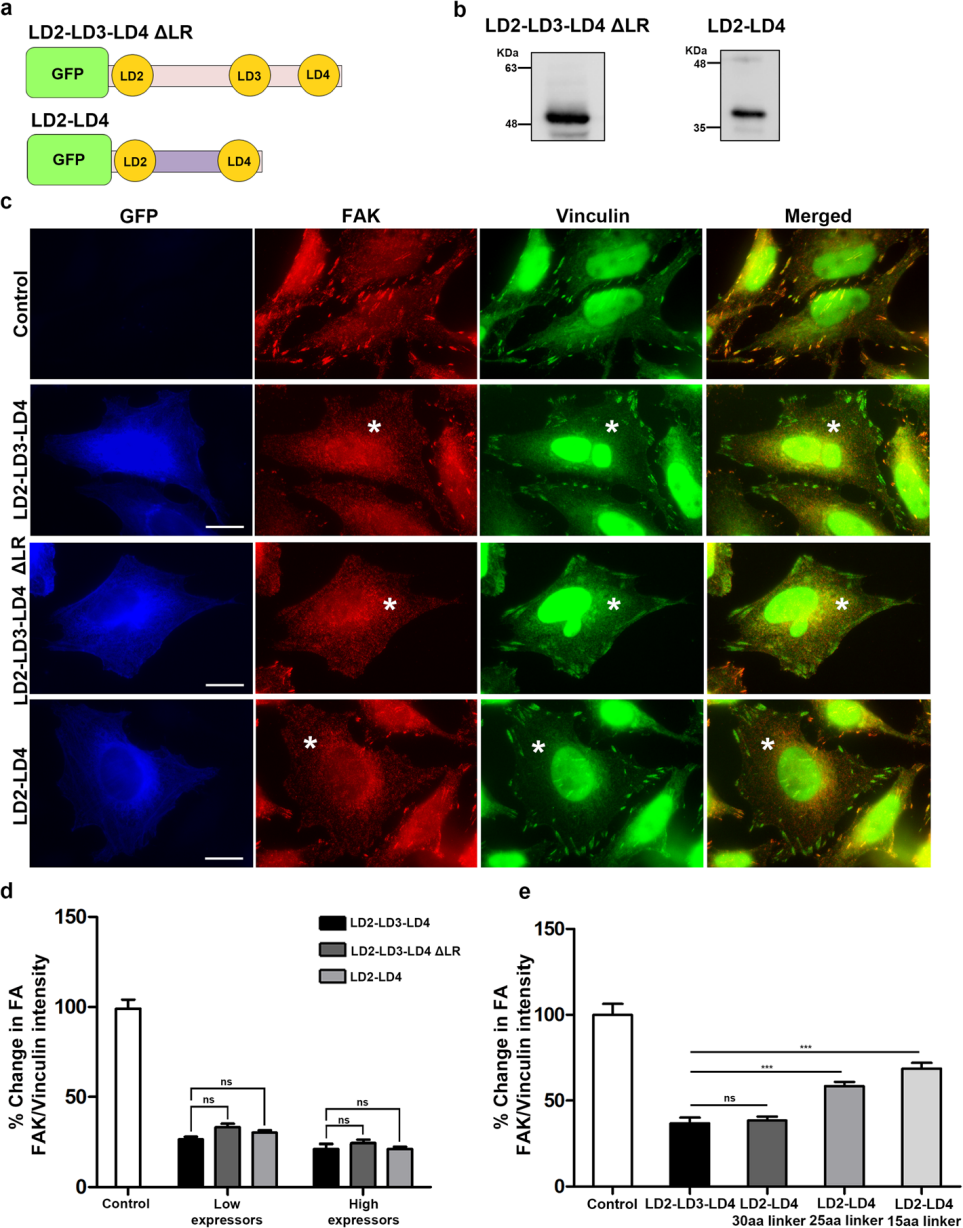
The LD2 and LD4 motifs of paxillin, are sufficient for displacement of FAK from FAs **a**) Schematic representation of LD2-LD3-LD4 ΔLR, composed of amino acids 139–279 of Paxillin fused to GFP; and LD2-LD4, composed of LD2 and LD4 motifs of paxillin, amino acids 139–162 and 261–279 respectively, joined together by a 30 amino acid-long flexible linker and fused to GFP. **b**) Representative Western Blot showing expression of stable proteins, encoding GFP fused LD2-LD3-LD4 ΔLR and LD2-LD4, in HeLa cells (expected molecular weight ~ 44 kDa and ~ 34 kDa respectively). **c**) Widefield images of HeLa cells, control or transiently transfected with GFP-fused LD2-LD3-LD4, LD2-LD3-LD4 ΔLR or LD2-LD4 and immunostained for FAK and Vinculin. Expressing cells are marked with an asterisk. Control cells display strong localization of FAK at FAs unlike cells expressing GFP-fused LD2-LD3-LD4, LD2-LD3-LD4 ΔLR or LD2-LD4. **d**) Quantification of the % change in the mean FAK/Vinculin intensity at FAs in control cells (100 ± 4.56, *n* = 335 FAs from control cells) and cells expressing GFP LD2-LD3-LD4 (26.33 ± 1.46, *n* = 163 FAs from 16 low-expressing and 21.2 ± 2.73, *n* = 147 FAs from 15 high-expressing cells), GFP LD2-LD3-LD4 ΔLR (33.04 ± 2, *n* = 150 FAs from 16 low-expressing and 24.32 ± 1.84, *n* = 133 FAs from 14 high-expressing cells) or GFP LD2-LD4 (30 ± 1.3, *n* = 140 FAs from 15 low-expressing and 21.22 ± 1.22, *n* = 225 FAs from 17 high-expressing cells). Both GFP LD2-LD3-LD4 ΔLR and GFP LD2-LD4 displace FAK from FAs in a dose dependent manner and as efficiently as GFP LD2-LD3-LD4 does. **e**) Quantification of the % change in the mean FAK/Vinculin intensity at FAs in control cells (100 ± 6.5, *n* = 119 FAs from 15 cells) and cells expressing GFP LD2-LD3-LD4 (36.65 ± 3.4, n = 140 FAs from 14 cells), or GFP LD2-LD4 with either a 30 amino acid linker (38.3 ± 2.35, *n* = 100 FAs from 14 cells), 25 amino acid linker (58.47 ± 2.44, *n* = 117 FAs from 15 cells) or a 15 amino acid linker (68.69 ± 3.31, *n* = 110 FAs from 15 cells) It is evident that the 30 amino acid long linker displays equivalent efficiency to displace FAK from FAs to LD2-LD3-LD4. Scale bars 10 μm. The error bars represent standard error of the mean (S.E.M). ***; *p* < 0.0001

We initially deleted the region upstream of LD2 (LD1-LD2 linking region-LR), previously reported to be necessary for optimal binding to FAK [23, 24, 30] and examined how it affected the peptide’s capacity to displace FAK from FAs (Fig. 7a). This construct led to expression of a stable polypeptide, at the expected molecular weight, hereunto referred as LD2-LD3-LD4 ΔLR (Fig. 7b). As shown, expression of LD2-LD3-LD4 ΔLR led to clear displacement of FAK from FAs, while Vinculin localization (used as an FA marker) was unaffected as expected (Fig. 7c). Quantification of the FAK to Vinculin ratio showed that LD2-LD3-LD4 ΔLR displaced FAK with the same efficiency as the original peptide, suggesting that the linking segment upstream of LD2 does not play a pivotal role for efficient binding of the LD2 and LD4 motifs to the FAT HPs in the cell (Fig. 7d).

Next, we went on to examine whether the intermediate linking region between LD2 and LD4, containing the LD3 motif, plays a role in the ability of the polypeptide to displace FAK. Using the DNA encoding for LD2-LD3-LD4 ΔLR as template, we replaced the region between the LD2 and LD4 motifs with a flexible standard linker (GGGGS). Optimization of the length of the linker was performed by evaluating the efficiency of LD2- GGGGSn-LD4 polypeptides, containing linkers of different sizes (15, 25 and 30 amino acids), to displace FAK from FAs (Fig. 7e). At the end, a 30 amino acid-long linker containing 6 (GGGGS) repeats was selected, leading to the generation of a new construct, hereunto referred to as LD2-LD4 (Fig. 7a and b). We went on to quantify the ability of LD2-LD4 to displace FAK from FAs in comparison to the original construct. As shown, LD2-LD4 is as efficient as the original in displacing FAK from FAs (Fig. 7c and d), suggesting that the sequence of the intermediate (LD2-LD4) linking region and LD3 are not essential for binding to FAK. The new polypeptide lacks critical phosphorylation sites present in the original and is devoid of any paxillin sequences other than the two LD motifs, ensuring improved specificity. Importantly the 30 amino acid linker is significantly shorter than the 99 amino acid linker contained in the original polypeptide; coupled with the removal of LD2 upstream sequences, this peptide is much smaller, only 69 amino acids (6 kDa), compared to the original peptide that was 226 amino acids (24 kDa) and thus well within the limits of solid-phase peptide synthesis. This effectively raises the possibility of using a synthetic polypeptide as an anti-metastatic agent.

### Inducible expression of LD2-LD4 interferes with the interaction of FAK with endogenous paxillin

**Fig. 8:**
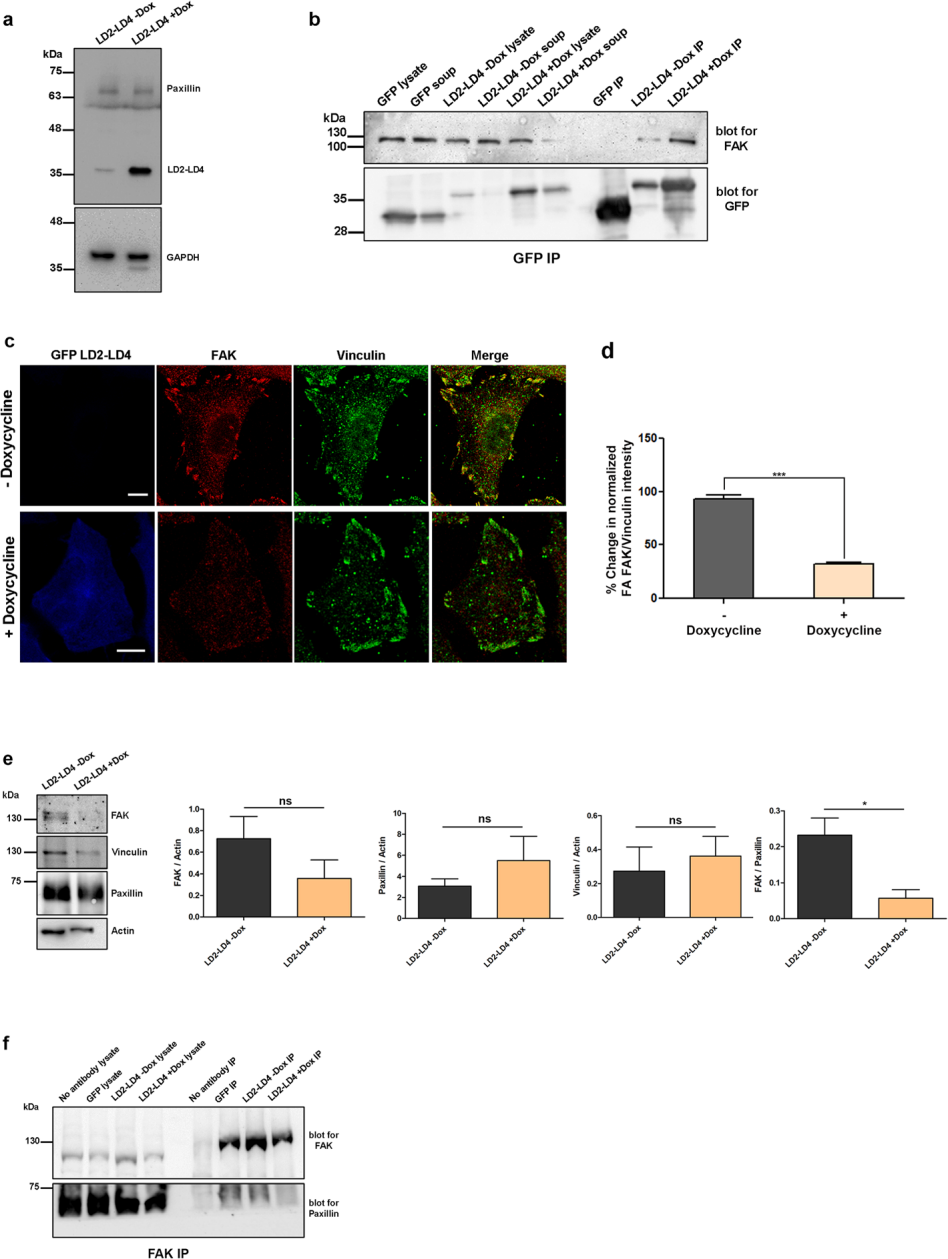
LD2-LD4 expression interferes with the interaction between FAK and paxillin. Representative Western Blot from inducible GFP LD2-LD4 stable Hela cells indicating the expression levels of GFP LD2-LD4 compared to endogenous paxillin, upon induction with 10 μg/ml doxycycline for 24 h. **b**) Western blot showing immunoprecipitated GFP, from Hela cells stably expressing GFP or GFP LD2-LD4 (from inducible GFP LD2-LD4 Hela cells, induced or not with 10 μg/ml doxycycline for 24 h), blotted for GFP and FAK. FAK co-precipitation is observed in cells inducibly expressing GFP LD2-LD4. **c**) Confocal images of PFA-fixed inducible GFP LD2-LD4 Hela cells, immunostained for FAK and Vinculin. Expression of GFP LD2-LD4 upon treatment with 10 μg/ml doxycycline for 24 h (bottom panel) leads to decrease in the intensity of FAK at FAs, unlike Vinculin. **d**) Quantification of the % change in the mean FAK/Vinculin intensity at FAs reveals ~ 67% reduction in the FA-localized FAK in cells inducibly expressing GFP LD2-LD4 (32.28 ± 1.664 *n* = 197 FAs from 27 cells) compared to control non-induced cells (100 ± 4.01 *n* = 211 FAs from 28 cells). **e**) Representative western blots from isolated FAs of inducible GFP LD2-LD4 Hela cells, non-induced (left lane) or induced with 10 μg/ml doxycycline for 24 h, blotted for FAK, Paxillin and Vinculin. Quantification of FAK/Actin (0.7252 ± 0.2068 in non-induced, 0.3583 ± 0.1684 in induced cells), Paxillin/Actin (3.075 ± 0.7076 in non-induced, 5.484 ± 2.322 in induced) and Vinculin/Actin (0.2735 ± 0.1407 in non-induced, 0.3610 ± 0.1156 in induced) ratios, show reduction of FAK at the FAs of induced cells, unlike Paxillin and Vinculin which display increased FA-localization, verifying the results of IF experiments. This is further confirmed by quantification of the normalized FAK/Paxillin ratio in induced (0.05682 ± 0.02397) and non-induced (0.2320 ± 0.04788) cells, indicating 75% reduction in FA-localized FAK in cells expressing GFP LD2-LD4. **f**) Western blot of immunoprecipitated FAK from induced (24 h treatment with 10 μg/ml doxycycline) and non-induced GFP LD2-LD4 Hela cells, blotted for FAK and paxillin. The amount of co-precipitated paxillin is reduced in induced cells expressing GFP LD2-LD4. Non-induced GFP LD2-LD4 cells incubated with no antibody were used as negative control. Scale bars 10 μm. The error bars represent standard error of the mean (S.E.M). *; *p* < 0.0.05

Having determined the minimum LD motif and linker length requirements of the polypeptide, we decided to confirm the molecular mechanism of action, which we postulated is the disruption of interactions between endogenous paxillin and FAK. Use of a transient expression system imposes limitations on using a biochemical approach, given the inability to attain 100% efficiency within a single transfection, uneven expression levels between transfected cells and variation in efficiency between transfections; we thus generated a stable HeLa cell line to inducibly express LD2-LD4 using a lentiviral vector system. As indicated in Fig. 8a, induction using Doxycycline, leads to the expression of a stable polypeptide at the expected molecular weight (~35kD), which interacts with FAK, similarly to the transiently expressed polypeptide (Fig. 8b). Furthermore, inducible expression of LD2-LD4 led to clear displacement of FAK from FAs, while Vinculin localization (used as an FA marker) was unaffected as expected (Fig. 8c). Quantification of the FAK to Vinculin ratio showed that the inducible expression of LD2-LD4 displaced FAK with the same efficiency as the transiently expressed peptide (Fig. 8d). To validate the IF results we performed biochemical fractionation to isolate FAs followed by Western blot analysis, so as to determine resident protein levels. As shown in Fig. 8e the levels of FAK at FAs are markedly reduced, upon inducible expression of the LD2-LD4 polypeptide, and this is further supported by quantification of FAK/Paxillin ratio (both normalized to actin expression levels) (Fig. 8e). In contrast, Vinculin and paxillin levels are increased, in agreement with the immunofluorescence results described above. Collectively, these results provide firm confirmation that LD2-LD4 expression displaces FAK from FAs.

As extensively discussed in previously published work (from our group and others), targeting of FAK to FAs depends on interaction of the LD motifs of endogenous paxillin with the FAT domain of FAK. We postulated that the overexpressed LD2-LD4 polypeptide, shown to bind FAK (Fig. 1d and S1e), interferes with the ability of FAK to bind endogenous paxillin and therefore prevents FA targeting. To confirm this, we performed co-immunoprecipitation experiments to isolate FAK and co-precipitated proteins, from extracts of induced Hela cells that stably express LD2-LD4 (uninduced Hela cells as well as GFP expressing cells were used as controls). As shown in Fig. 8f, the levels of co-precipitated Paxillin in induced cells are markedly reduced compared to control cells. These data clearly show that expression of the polypeptide disrupts the interaction of FAK with endogenous paxillin, thus confirming the molecular mechanism of action.

### Dimers of a single LD motif can effectively displace FAK from FAs and reduce tumor cell migration

The polypeptide used throughout this study consists of two separate LD motifs that have different sequences. However, designing and delivering a small molecule inhibitor consisting of two molecules would be quite challenging and complicated. We thus went on to examine the possibility that a single LD motif could bind to both HPs of the FAT domain and displace FAK.

**Fig. 9:**
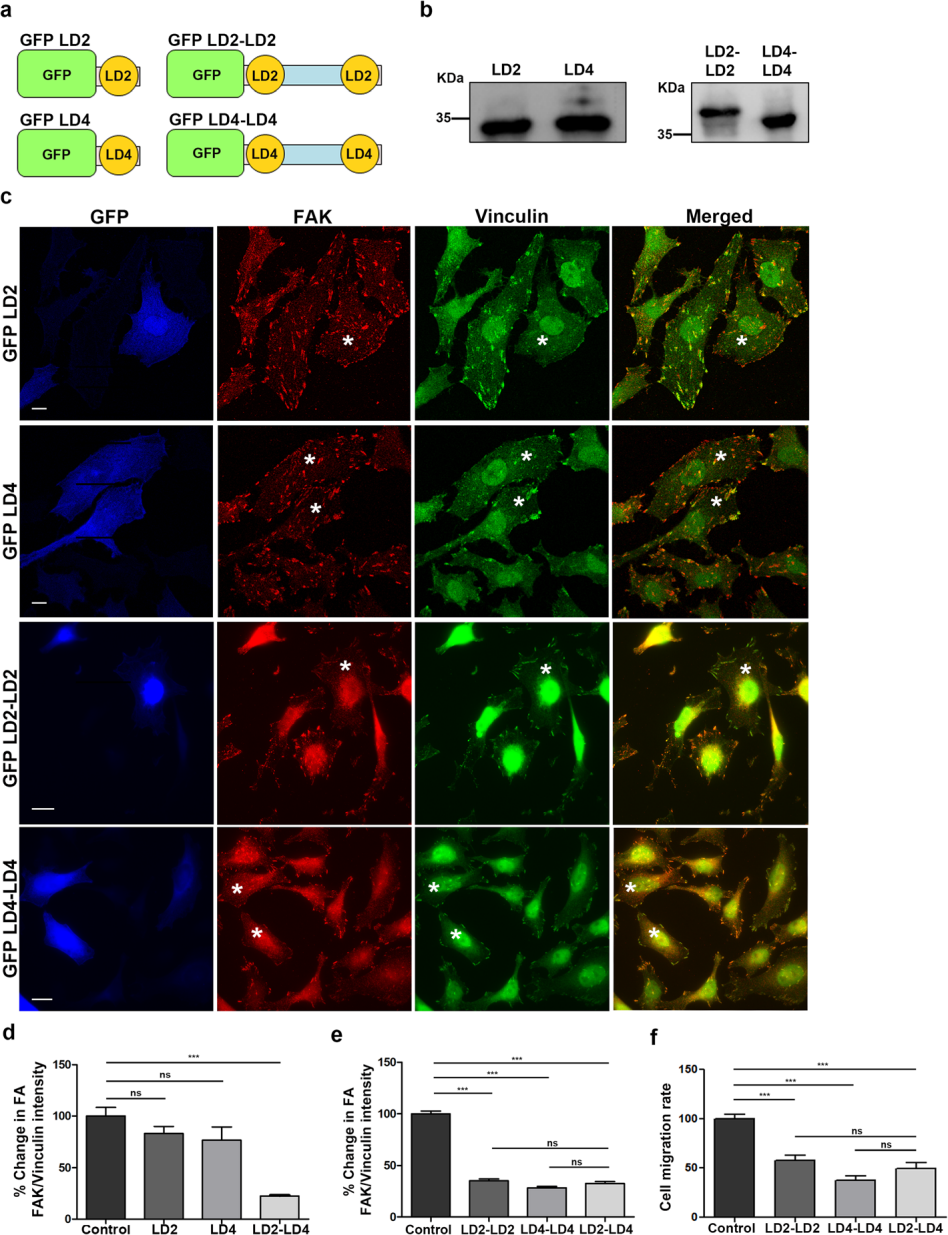
Both FAK HPs can be effectively targeted with a single LD motif in the form of a dimer. **a**) Schematic representation of LD2 (composed of 420–440 amino acids of paxillin), LD4 (composed of 783–845 amino acids of paxillin), LD2-LD2 (composed of two LD2 motifs linked together through a flexible 30 amino acid linker) and LD4-LD4 (composed of two LD4 motifs linked together through a flexible 30 amino acid linker) constructs, fused to GFP. **b**) Representative Western Blots showing expression of stable proteins encoding GFP fused LD2 and LD4 (expected molecular weight ~ 31 kDa) in the left panel and GFP fused LD2-LD2 and LD4-LD4 (expected molecular weight ~ 35 kDa) in the right panel. **c**) Widefield images of HeLa cells, transiently transfected with GFP-fused LD2, LD4, LD2-LD2 or LD4-LD4 and immunostained for FAK and Vinculin. Expressing cells are marked with an asterisk. GFP LD2 and GFP LD4 expressing cells display strong localization of FAK at FAs, similarly to control cells. GFP LD2-LD2 and GFP LD4-LD4 expressing display reduction in FA-localized FAK, compared to control cells. **d**) Quantification of the % change in the mean FAK/Vinculin intensity at FAs in control cells (100 ± 8.43, *n* = 388 FAs from 25 cells) and cells expressing GFP LD2 (83 ± 6.97, *n* = 276 FAs from 25 cells), GFP LD4 (76.54 ± 12.88, *n* = 441 FAs from 32 cells) or GFP LD2-LD4 (22.51 ± 0.9, *n* = 406 FAs from 30 cells). Neither GFP LD2 nor GFP LD4 displace FAK from FAs, unlike GFP LD2-LD4. **e**) Quantification of the % change in the mean FAK/Vinculin intensity at FAs in control cells (100 ± 2.64, *n* = 542 FAs from 30 cells) and cells expressing GFP LD2-LD2 (35.18 ± 1.75, *n* = 334 FAs from 27 cells), GFP LD4-LD4 (28.37 ± 1.31, *n* = 322 FAs from 28 cells) or GFP LD2-LD4 (32.61 ± 1.76, n = 276 FAs from 27 cells). Both GFP LD2-LD2 and GFP LD4-LD4 displace FAK from FAs, as efficiently as GFP LD2-LD4. **f**) Expression of GFP LD2-LD2 or GFP LD4-LD4 leads to decrease in the migration rate of MDA MB-231 cells (57.99 ± 4.87, *n* = 73 cells expressing GFP LD2-LD4 and 37.52 ± 4.54, n = 33 cells expressing GFP LD4-LD4, compared to 100.0 ± 4.038, *n* = 89 control cells), similarly to GFP LD2-LD4 does (49.84 ± 5.43, *n* = 49 cells). Scale bars: 10 μm. The error bars represent standard error of the mean (S.E.M). ***; *p* < 0.0001

We therefore proceeded to generate two new constructs encoding either LD2 or LD4 fused to GFP, hereunto referred to as GFP LD2 and GFP LD4 respectively (Fig. 9a) and examined the efficiency of the stable polypeptides expressed (Fig. 9b), to displace FAK from FAs. LD2 has been shown to bind to both HPs with equally high affinity, whereas LD4 only binds HP1 with high affinity, thus we expected that GFP LD2 would be more efficient in displacing FAK compared to GFP LD4 [43, 58, 59]. However, both LD2 and LD4 as monomers failed to displace FAK from FAs (Fig. 9 c and d). These results, are in agreement with previous studies showing that peptides containing both LD2 and LD4 display higher affinity for FAT compared to single LDs [31, 43, 58, 60], as well as the results discussed earlier, showing that LD2 and LD4 have to be connected through a flexible linker of the proper size in order to displace FAK from FAs. Taken together, these data provide strong evidence that an LD motif dimer is required for a high affinity interaction with FAK to take place.

To test this possibility and at the same time determine if a single LD motif could in fact target both HPs, we generated two new constructs encoding LD2-LD2 or LD4-LD4, separated by the optimized 30 amino acid linker described earlier (Fig. 9a and b). As shown (Fig. 9c-e) both LD2-LD2 and LD4-LD4 effectively displace FAK from FAs, with a similar efficiency as LD2-LD4 suggesting that both LD2 and LD4 can bind both HPs. Importantly, expression of either polypeptide reduced the migratory capacity of MDA MB-231 cells, as efficiently as LD2-LD4 (Fig. 9f).

These results lead to the conclusion that the function of FAK, specifically at FAs, can be efficiently targeted using the above described strategy, as long as the peptide topology is maintained (two LD motifs connected with an appropriate linker). More importantly, it suggests that a single small molecule mimic could potentially bind both HPs eliminating the need for two individual molecules.

## Discussion

Both kinase-dependent and kinase-independent scaffolding functions of FAK are implicated in tumor development and metastasis and have previously been targeted, separately, by specific inhibitors. In this study, we present a novel strategy that can target and effectively block both adaptor and enzymatic functions of FAK at FAs, the major sites of FAK activity. The strategy relies on competing with interactions with physiological binding partners essential for FAK’s FA localization, effectively displacing FAK from these complexes. We generated a polypeptide containing the Paxillin LD2-LD3-LD4 motifs, which binds two hydrophobic pockets within the FAK FAT domain, thus preventing interactions with paxillin, previously described to be necessary for FA localization. Given that the polypeptide lacks the Paxillin localization determinants (LIM domains) it promotes FAK displacement from FAs. This is, to our knowledge, the first time an exogenously introduced molecule, is shown to prevent FAK FA localization, in a controlled, dose-dependent manner.

In contrast, when we tested C4, an inhibitor designed to block FAK-VEGFR3 interactions and reported to displace FAK from FAs [17, 47], we found that it failed to interfere with FA targeting of FAK, even at high concentrations. Given previous work showing that C4 acts through interactions with His 1025 on Helix 4 of the FAK FAT domain, we hypothesized that it may sterically hinder access to HP1. However, our data suggest that this inhibitor might not prevent interactions at HP1 or that interactions at both HPs need to be blocked, in order to displace FAK from FAs. This was also suggested by previous work showing that FAK mutants in which paxillin binding is completely abrogated by disruption of both HPs cannot localize to focal adhesions whereas FAK mutants that retain at least one functional HP (either HP1 or HP2), can still be successfully targeted to focal adhesions [33, 43].

Despite effective FAK displacement from FAs, localization of core FA proteins, including Talin, Integrins, Vinculin and Tensin was unaffected, suggesting that LD2-LD3-LD4 effects are FAK-specific, leaving FA composition broadly unchanged. It is important to note that these effects are observed on mature FAs and not nascent adhesions, where FAK has been proposed to promote the recruitment of talin [61]. Possible elimination of Talin from nascent adhesions by LD2-LD3-LD4 could potentially contribute to the migration defects observed. More importantly, the LD2-LD3-LD4 induced displacement of FAK from FAs has clear consequences on both FAK’s kinase-dependent functions, including activation and downstream integrin signaling, as well as kinase-independent scaffolding functions. Firstly, we show significant reduction of FAK phosphorylation at both Tyr 397 and Tyr576. Since autophosphorylation of Tyr397 is a direct consequence of FAK clustering within FA complexes [62] and given that LD2-LD3-LD4 displaces FAK from FAs, autophosphorylation is significantly reduced. Consequently, unphosphorylated Tyr397 can no longer support Src binding leading to reduced Src recruitment at FAs and Tyr576 phosphorylation [37, 50, 63, 64]. In addition, expression of LD2-LD3-LD4 leads to reduced Paxillin phosphorylation, one of the major FAK/Src downstream targets, but also to dramatically reduced levels of total phosphotyrosine at FAs. Tyrosine phosphorylation is the major signal transduction mechanism from FAs, therefore the observed reduction, suggests that LD2-LD3-LD4 expression, blocks integrin signaling via displacement of FAK from FA complexes and inhibition of downstream target phosphorylation. Therefore, LD2-LD3-LD4 can efficiently inhibit FAK’s enzymatic activity and downstream signal transduction events without directly targeting its catalytic domain; instead it competes with endogenous paxillin, thus preventing FAK’s targeting to FAs, which is essential for activation. Moreover, the LD2-LD3-LD4 induced displacement of FAK from FAs, also displaces p130Cas, an adaptor protein recruited to these multi-protein complexes through FAK. This suggests that our approach also targets and inhibits FAK’s kinase-independent scaffolding functions, providing a distinct advantage over existing inhibitors.

FAK has a prominent role in FA assembly and disassembly, processes inherently linked to cell migration and metastasis. Expression of LD2-LD3-LD4 leads to significant defects in cell spreading and FA turnover, resembling the FAK-null fibroblast phenotype [53]. Consequently, we observed a clear, dose-dependent inhibition of 2D cell migration, suggesting that our approach effectively blocked FAK activity, in promoting cell movement. Despite the aforementioned defects, LD2-LD3-LD4 was well-tolerated, even when highly expressed, suggesting that binding is specific and non-toxic. This is yet another advantage over existing FAK kinase inhibitors, which have raised concerns with respect to toxicity due to limited specificity.

Perhaps more importantly than inhibiting cell migration, LD2-LD3-LD4 dramatically reduced the capacity of the highly metastatic MDA MB-231 cells to invade, in gel invasion assays. This suggests that this strategy for FAK inhibition has the potential to be further developed into an anti-metastatic agent, provided that efficient delivery can be accomplished. Our work points towards this direction, given that the only requirement to achieve effective FAK displacement from FAs is a 6kD polypeptide, comprised of an LD motif dimer (either LD2 or LD4 or a combination of the two), linked through a flexible, 30 amino-acid linker.

Current work focuses on a preclinical study to evaluate the strategy in vivo*,* using a mouse solid tumor model, as well as testing of synthetic peptide analogs and in silico molecular docking screens, to identify small molecule mimics capable of binding both HPs with high affinity. In addition, given the fact that both LD2 and LD4 can efficiently bind both HPs of the FAT domain of FAK (under the conditions described earlier), molecular dynamic simulations are currently under way, for the determination of an optimized sequence that could bind both HP1 and HP2 with improved affinity, that would possibly allow its use as a monomer.

The precise mechanism through which FAK is displaced from FAs in the presence of the peptide is not entirely clear, but several lines of evidence suggest that the two LD motifs on each polypeptide engage an individual FAT HP site on a single FAK molecule. Furthermore, the fact that the two LD motifs are linked probably enhances the avidity for this type of simultaneous binding. However, the possibility that the tandem LD motifs on the polypeptide link FAT domains from distinct FAK molecules, thus sequestering them away from FAs, cannot be excluded. This type of interaction is nonetheless highly unlikely given that previous studies using X-ray crystallography, solution NMR, and homology modeling, have shown the FAK-paxillin interaction to be mediated through binding of the LD2 and LD4 motifs of a single paxillin molecule to a single FAT domain [58]. In addition, our results, showing that for effective FAK displacement from FAs it is imperative that the two LD motifs of the polypeptide are linked through a flexible linker of a specific minimum length of 30 amino acids (shorter linkers of 15, 20 and 25 amino acids proved ineffective), also suggest that each polypeptide binds one FAK molecule. This experimentally determined optimal linker length is equivalent to ~ 90 Å, and thus satisfies the requirement for sufficient length between two tandem LDs, so that the peptide can wrap around the FAT domain and allow simultaneous binding to the HPs of a single FAK molecule, in a parallel orientation, as previously proposed [58].

## Conclusion

In conclusion, our data show that LD2-LD4 is a novel FAK inhibitor, that is well tolerated and functions by displacing the protein from FAs. This, as expected, results in the site-specific inhibition of FAK’s kinase and scaffolding activities. Importantly, this peptide leads to impaired cell spreading, migration and invasion, raising the possibility that this promising new strategy can form the basis for the design of effective small-molecule FAK inhibitors, to prevent tumor metastasis.

## Supplementary Information

**Additional file 1: Table S1.** List of primers used for construct generation.

**Additional file 2: Fig. S1.** LD2-LD3-LD4 displaces both endogenous and exogenous FAK from FAs. **Fig. S2.** Inhibition of FAK Tyr397 phosphorylation prevents further activation of FAK without affecting its localization at FAs. **Fig. S3.** LD2-LD3-LD4 expression leads to displacement of FAK from FAs and reduction of the migratory capacity of tumor cells.

## Data Availability

The data used and analyzed for the current study are available within the manuscript and its supplemental information files.

## Abbreviations

FAK: Focal Adhesion Kinase
FAs: Focal Adhesions
FAT: Focal Adhesion Targeting
ECM: Extracellular Matrix
MMPs: Matrix Metalloproteinases
VEGFR: Vascular Endothelial Growth Factor Receptor-3
IGFR: Insulin-like Growth Factor Receptor
Mdm2: Mouse Double Minute 2
PI3K: Phosphoinositide 3 Kinase
HPs: Hydrophobic Pockets
LIM: Lin11, Isl-1 & Mec-3
FRET: Förster Resonance Energy Transfer
2-D: 2-Dimensional
3-D: 3-Dimensional
