# Supplementary material for: FAK displacement from focal adhesions: a promising strategy to target processes implicated in cancer progression and metastasis
Source: Cell Commun Signal. 2021 Jan 7;19:3. doi: 10.1186/s12964-020-00671-1 (PMC7791867; doi:10.1186/s12964-020-00671-1)
Supplement: Supplementary file 4 — Additional file 1: Table S1. List of primers used for construct generation. [file 12964_2020_671_MOESM2_ESM.pdf]

Supplementary Table S1

Primer Name	Primer Sequence
F1	GCGGCCGCtccagtgaggccctaaatggcactgtgattg
F2	aaGCGGCCGCGtccagtgaggccctaaatggcactgtgattg
F3	TGGTGGTGGTGGTGGTggcagcaacctctcggaactggaca
F4	aaGCGGCCGCGGTGGTGGTGGTGGTGGTGGTGGT
F5	GGCGGCAGCGGCGGCGGCaacctctcggaactggac
F6	GGCGGCAGCGGCGGCGGCgctacacgagaactgt
F7	Gcggctggttccggagagtttggcagcaacctctcggaact
F8	aaTCTAGAgggtcagcggggtcagcggctggttccggagagttt
F9	TGGTGGTGGTtcagctacacgagaactgga
F10	aaGCGGCCGCGGTGGTGGTGGTGGTGGTGGTGGTtcagctacac
F11	aagaattcatggtgagcaagggcgaggagctgttca
R1	aaCTCGAGCTAgaacttaaagtcagagagggacgccatcagc
R2	aaGAATTCCTAgaacttaaagtcagagagggacgccatcagc
R3	aaCTCGAGCTAgaacttaaagtcagagagggacgccatcagctcatccagttctcgtgtagc
R4	gccagagccacctccgccgccactggggggattatgttgaacagc
R5	gagccacctccgccagagccacctccgccagagccacctccgccagagccacctccgcC
R6	tcgtgtagctgaagagccacctccgccagagccacctccgccagagccacctccgccagag
R7	ccagttctcgtgtagctgaagagccacctccgccagagccacctccgccagagccacctc
R8	ctcatccagttctcgtgtagctgaagagccacctccgcCagagccacctccgccagagc
R9	aaCTCGAGTTAggggggattatgttgaacagcattca
R10	aaCTCGAGTTActgtgccatgaacttaaagtcagaga
R11	gccggctgcgcttcctgcgcttccgccactggggggattatgttgaacag
R12	aaTCTAGAagaacctccgccgaattctccactgccggctgcgcttcctgcgcttcc
R13	aaCTCGAGCTAtgctgagaagccactggggggattatgttgaacagcattcagttcc
R14	Ctgcgcttccgtcagagagggacgccatca
R15	tccactgccggctgcgcttcctgcgcttccgtcagagagg
R16	gggaattcctcgagctagaacttaaagtca
