# Supplementary material for: FAK displacement from focal adhesions: a promising strategy to target processes implicated in cancer progression and metastasis
Source: Cell Commun Signal. 2021 Jan 7;19:3. doi: 10.1186/s12964-020-00671-1 (PMC7791867; doi:10.1186/s12964-020-00671-1)
Supplement: Supplementary file 5 — Additional file 2: Fig. S1. LD2-LD3-LD4 displaces both endogenous and exogenous FAK from FAs. Fig. S2. Inhibition of FAK Tyr397 phosphorylation prevents further activation of FAK without affecting its localization at FAs. Fig. S3. LD2-LD3-LD4 expression leads to displacement of FAK from FAs and reduction of the migratory capacity of tumor cells. [file 12964_2020_671_MOESM3_ESM.pdf]

Additional File 2

FAK displacement from focal adhesions: A promising strategy to target processes implicated in cancer progression and metastasis

Ioanna Antoniades†, Maria Kyriakou†, Anna Charalambous, Katerina Kalalidou, Andri Christodoulou, Maria Christoforou and Paris A. Skourides*

Supplementary Figures

Figure S1:

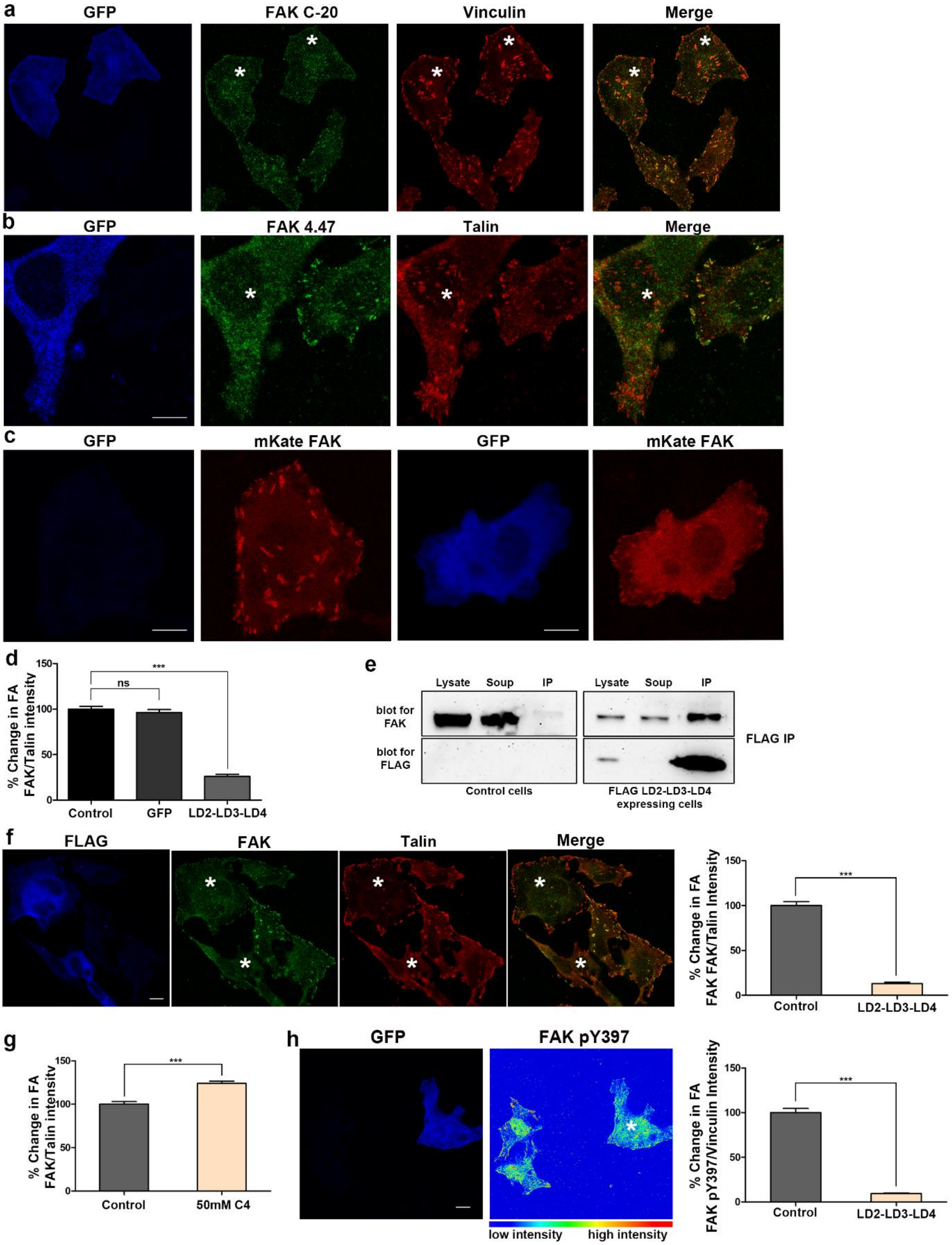

Fig. S1: LD2-LD3-LD4 displaces both endogenous and exogenous FAK from FAs

a-b) Confocal images of HeLa cells transfected with GFP LD2-LD3-LD4 and immunostained for FAK and Vinculin (a) or FAK and Talin (b), using two different antibodies against FAK. Staining with both antibodies shows clear displacement of FAK in cells expressing GFP LD2-LD3-LD4 (marked with asterisk). c) Confocal images of live HeLa cells expressing mKate FAK alone (1st and 2nd panel) or in combination with GFP LD2-LD3-LD4 (3rd and 4th panel). Localization of over-expressed FAK at FAs is less efficient in cells expressing GFP LD2-LD3-LD4. d) Quantification of the % change in the mean FAK/Talin intensity, indicating that the localization of FAK at FAs is not affected by the transfection and expression of GFP (100 ± 2.95, n=150 FAs from 17 control cells; 96.33 ± 3.19, n=154 FAs from 15 GFP expressing cells; 26.12 ± 2.28, n=217 FAs from 17 LD2-LD3-LD4 expressing cells) . e) Western blots showing immunoprecipitated FLAG-tagged LD2-LD3-LD4, blotted for FLAG and FAK. Co-precipitation of FAK (125kDa) is only observed in HeLa cells expressing FLAG LD2-LD3-LD4 (marked with asterisk). f) Confocal images and quantification of % change in the mean FAK/talin intensity at FAs, in control and FLAG LD2-LD3-LD4 expressing cells, fixed with Methanol/Acetone and immunostained for FLAG and Talin. Expression of LD2-LD3-LD4 leads to decrease in the FAK/Talin ratio compared to control cells (100 ± 4.33, n=305 FAs from 25 control cells, 13.04 ± 1.47, n=272 FAs from 28 LD2-LD3-LD4 expressing). g) Quantification of the % change in the mean FAK/Talin intensity in control and C4-inhibitor treated cells reveals that C4 does not block localization of FAK at FAs (100 ± 2.91, n=66 FAs from 10 control cells, compared to 123.9 ± 2.69, n=108 FAs from 10 C4 treated cells). h) Confocal images and quantification of the % change in the mean FAK pTyr397/Vinculin intensity at FAs, in control and GFP LD2-LD3-LD4 expressing cells, fixed with PFA and immunostained against phosphorylated FAK (pTyr397) and Vinculin. Signal is shown intensity coded. A dramatic reduction in the phosphorylated FAK/Vinculin ratio, is observed in LD2-LD3-LD4 expressing cells (100 ± 4.72, n=357 FAs from 30 cells; 9.38 ± 0.52, n=459 FAs from 30 expressing cells). Scale bars: 10 µm. The error bars represent standard error of the mean (S.E.M). ***; $p<0.0001$

Figure S2:

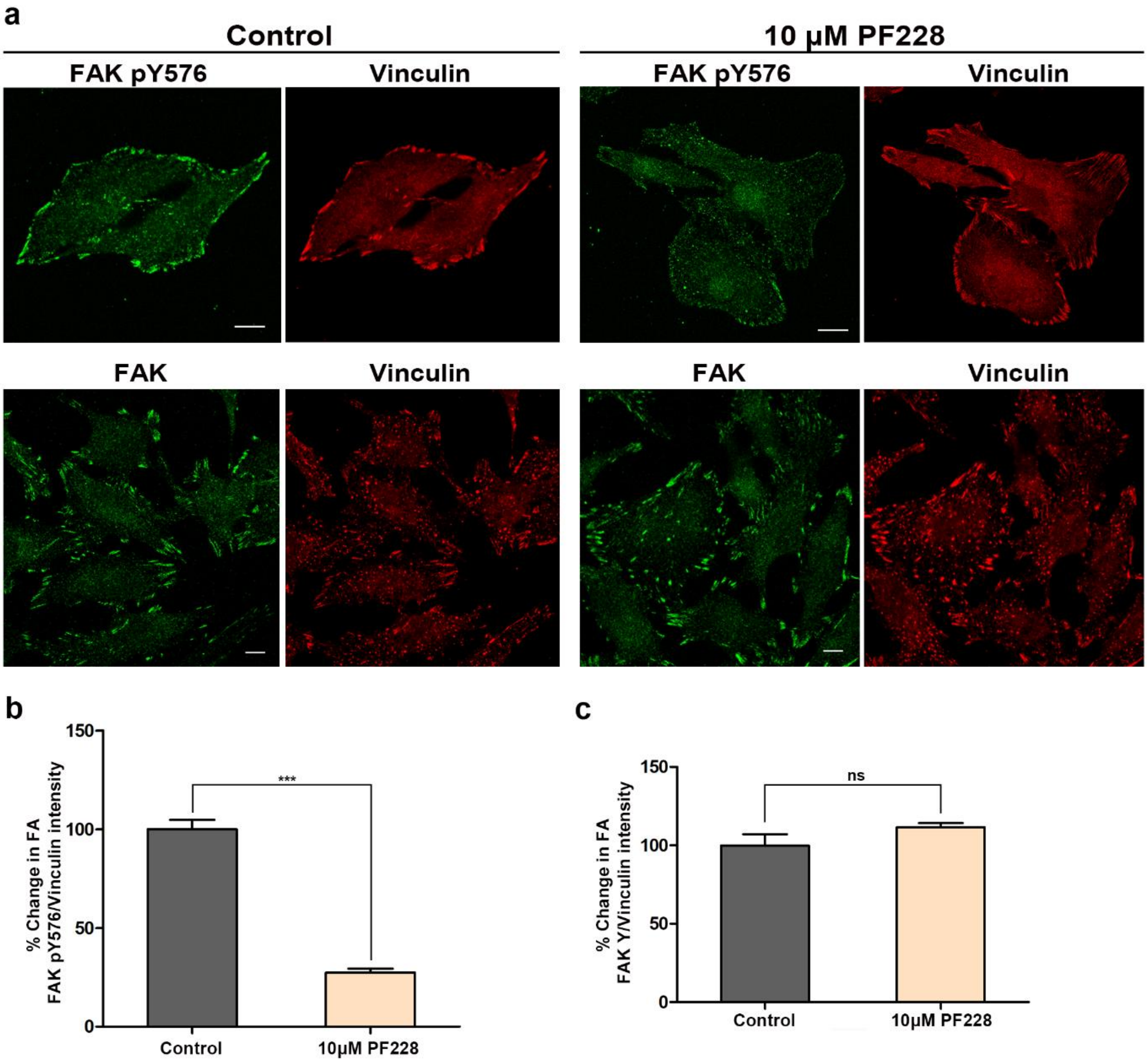

Fig. S2: Inhibition of FAK Tyr397 phosphorylation prevents further activation of FAK without affecting its localization at FAs a) Confocal images of control cells and cells treated with the PF228 FAK inhibitor, fixed with PFA and immunostained against phosphorylated (pTyr576) (in top panel), or total FAK (in bottom panel) and Vinculin. b-c) Treatment with the inhibitor leads to reduced phosphorylation on Tyr576 as indicated by the quantification of the % change in the mean pFAK Tyr576/Vinculin intensity (b) (27.36 ± 2.09, n=92 FAs from 10 PF-228 treated cells, compared to 100 ± 4.79, n=98 FAs from 10 control cells), without affecting the localization of FAK at FAs, as indicated by the quantification of the FAK/Vinculin intensity at FAs (c) (111.6 ± 2.59, n=296 FAs from 25 PF-228 treated cells, compared to 100 ± 7.06, n=308 FAs from 25 control cells). Scale bars: 10 µm. The error bars represent standard error of the mean (S.E.M). ***; p<0.0001

Figure S3:

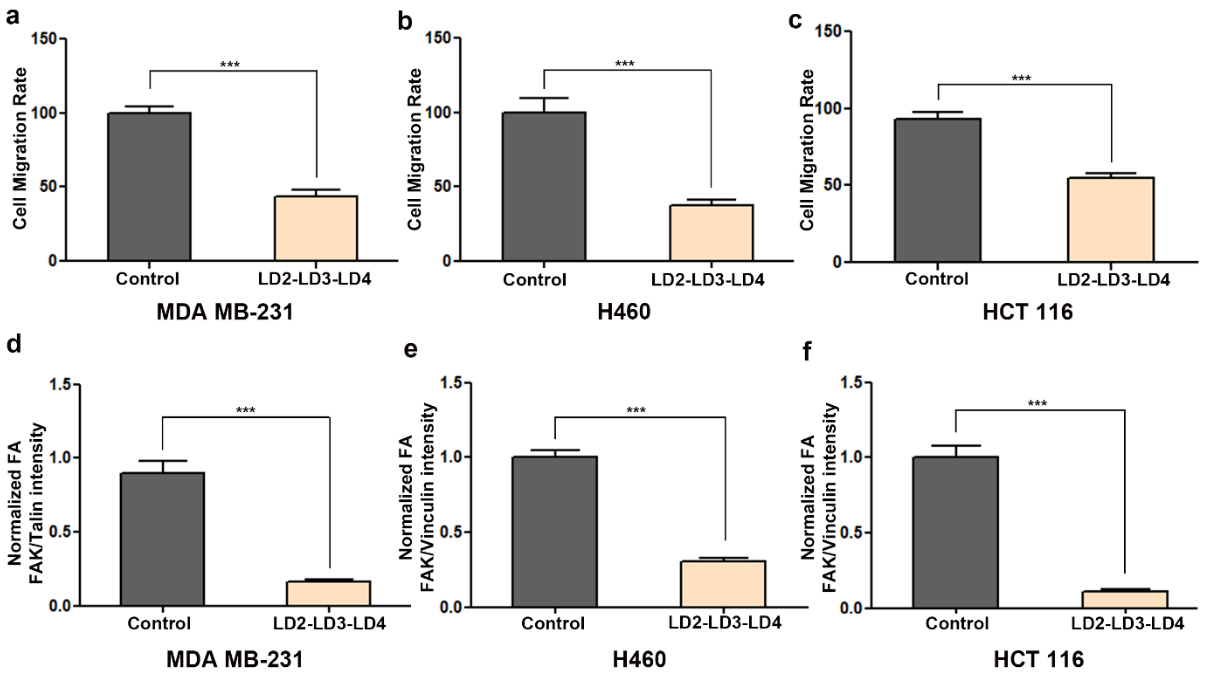

Fig. S3: LD2-LD3-LD4 expression leads to displacement of FAK from FAs and reduction of the migratory capacity of tumor cells

(A-C) Expression of GFP LD2-LD3-LD4 leads to decrease in the migration rate of (A) MDA MB-231 (43.55 ± 4.2 in expressing cells, n=41 cells compared to 100 ± 4.0 in control cells, n=89 cells), (B) H460 (37.31 ± 4.37 in expressing cells, n=27 cells compared to 99.99 ± 9.67 in control cells, n=31 cells) and (C) HCT 116 cells (54,63 ± 3.5, n=109 expressor cells compared to 92.77 ± 4.75, n=77 control cells). (D) Quantification of the mean FAK/Talin intensity ratio in MDA-MB231 cells transfected with GFP LD2-LD3-LD4, indicates efficient displacement of FAK from FAs (0.16 ± 0.017, n=47 FAs, in GFP LD2-LD3-LD4 expressing cells compared to 0.9 ± 0.08, n=71 FAs, in control cells). (E-F) Quantification of the mean FAK/Vinculin intensity ratio in H460 (E) and HCT 116 (F) cells transfected with GFP LD2-LD3-LD4. Expression of GFP LD2-LD3-LD4 leads to reduction of FAK/Vinculin ratio (0.31 ± 0.018, n=217 FAs in expressing cells compared to 1 ± 0.04, n=228 FAs in control H460 cells; 0.11 ± 0.018, n=53 FAs in expressing cells compared to 1 ± 0.08, n=65 FAs in control HCT 116 cells). The error bars represent standard error of the mean (S.E.M). ***; p<0.0001
